# Evolution-Operator-Based Single-Step Method for Image Processing

**DOI:** 10.1155/IJBI/2006/83847

**Published:** 2006-02-02

**Authors:** Yuhui Sun, Peiru Wu, G. W. Wei, Ge Wang

**Affiliations:** ^1^ Department of Mathematics, College of Natural Science, Michigan State University, MI 48824, USA; ^2^ Department of Electrical and Computer Engineering, College of Engineering, Michigan State University, MI 48824-1226, USA; ^3^ Department of Radiology and Department of Biomedical Engineering, University of Iowa, Iowa City, IA 52242, USA

## Abstract

This work proposes an evolution-operator-based single-time-step method for image and signal processing. The key component of the proposed method is a local spectral evolution kernel (LSEK) that analytically integrates a class of evolution partial differential equations (PDEs). From the point of view PDEs, the LSEK provides the analytical solution in a single time step, and is of spectral accuracy, free of instability constraint. From the point of image/signal processing, the LSEK gives rise to a family of lowpass filters. These filters contain controllable time delay and amplitude scaling. The new evolution operator-based method is constructed by pointwise adaptation of anisotropy to the coefficients of the LSEK. The Perona-Malik-type of anisotropic diffusion schemes is incorporated in the LSEK for image denoising. A forward-backward diffusion process is adopted to the LSEK for image deblurring or sharpening. A coupled PDE system is modified for image edge detection. The resulting image edge is utilized for image enhancement. Extensive computer experiments are carried out to demonstrate the performance of the proposed method. The major advantages of the proposed method are its single-step solution and readiness for multidimensional data analysis.


Hindawi Publishing Corporation
International Journal of Biomedical Imaging
Volume 2006, Article ID 83847, Pages 1–27
DOI 10.1155/IJBI/2006/83847

### Evolution-Operator-Based Single-Step Method for Image Processing

**Yuhui Sun,[1] Peiru Wu,[1] G. W. Wei,[1,2] and Ge Wang[3]**

[1] *Department of Mathematics, College of Natural Science, Michigan State University, MI 48824, USA*
[2] *Department of Electrical and Computer Engineering, College of Engineering, Michigan State University, MI 48824-1226, USA*
[3] *Department of Radiology and Department of Biomedical Engineering, University of Iowa, Iowa City, IA 52242, USA*

Received 28 July 2005; Revised 27 October 2005; Accepted 7 November 2005
Recommended for Publication by Sun Yoo

This work proposes an evolution-operator-based single-time-step method for image and signal processing. The key component of the proposed method is a local spectral evolution kernel (LSEK) that analytically integrates a class of evolution partial differential equations (PDEs). From the point of view PDEs, the LSEK provides the analytical solution in a single time step, and is of spectral accuracy, free of instability constraint. From the point of image/signal processing, the LSEK gives rise to a family of lowpass filters. These filters contain controllable time delay and amplitude scaling. The new evolution operator-based method is constructed by pointwise adaptation of anisotropy to the coefficients of the LSEK. The Perona-Malik-type of anisotropic diffusion schemes is incorporated in the LSEK for image denoising. A forward-backward diffusion process is adopted to the LSEK for image deblurring or sharpening. A coupled PDE system is modified for image edge detection. The resulting image edge is utilized for image enhancement. Extensive computer experiments are carried out to demonstrate the performance of the proposed method. The major advantages of the proposed method are its single-step solution and readiness for multidimensional data analysis.

Copyright © 2006 Yuhui Sun et al. This is an open access article distributed under the Creative Commons Attribution License, which permits unrestricted use, distribution, and reproduction in any medium, provided the original work is properly cited.

### 1. INTRODUCTION

Image denoising, restoration, edge detection, and enhancement are fundamental problems in image processing, computer vision, and artificial intelligence [1]. The solution to these problems is crucial to automatic control, robotics, imaging, target tracking, and telecommunication. A variety of methods have been proposed to tackle these problems in the past few decades. Among these methods, partial-differential-equation- (PDE-) based methods have recently received much attention. Witkin [2] introduced a parabolic evolution equation (i.e., the heat equation) for image denoising. The basic idea behind Witkin's approach is to evolve an original image under a diffusion process so as to effectively remove noise in the image surface. Such process is formally equivalent to the standard Gaussian filter. His approach has been intensively studied in terms of the scale-space process [3–5]. Based on a discrete version of the diffusion equation

$$u_j^{s+1} = u_j^s + R\left(u_{j-1}^s - 2u_j^s + u_{j+1}^s\right),$$

$$u_j^0 = u_j(t_1), \quad j = 1, \ldots, N, \; s = 0, 1, \ldots, S, \tag{1}$$

where

$$R = b\Delta t, \qquad S = \frac{(t_2 - t_1)}{\Delta t}, \tag{2}$$

Wei and Zhao showed that [6] the effect of the diffusion after $S$ iterations can be expressed as a convolution

$$u_j^S = \sum_{k=j-S}^{j+S} W(k, S) u_k(t_1), \tag{3}$$

where the filter $W(k, S)$ can be explicitly given by

$$W(k,S) = \begin{cases} \displaystyle\sum_{h=0}^{(S-k)/2} g(k, S, 2h), \; \forall S-k \text{ even}, \\ \displaystyle\sum_{h=1}^{(S-k+1)/2} g(k, S, 2h-1), \; \forall S-k \text{ odd}, \end{cases} \tag{4}$$

$$g(k, S, h) = \frac{S! R^{S-h} (1-2R)^h}{((S+k-h)/2)!((S-k-h)/2)!h!}. \tag{5}$$

Therefore, the result of the multiple-step time evolution described in discrete diffusion equation (1) can be exactly obtained via the single-step convolution (3). It can be verified that

$$\sum_{k=j-S}^{j+S} W(k,S) = 1, \qquad W(-k,S) = W(k,S), \quad \forall k = 1,\ldots,S. \tag{6}$$

A two-dimensional (2D) generalization of (3) is straightforward. Wei and Zhao also showed that $W(k,S)$ is a generalization of the Hanning filter [7] and applied it to chaos synchronization [6] and trend estimation [8].

Like the Gaussian filter, the diffusion process not only removes noise, but also smears image edges and leads to poor visual effects. To address such an issue, Perona and Malik proposed anisotropic diffusion process [9] in which the diffusion coefficient is replaced by a function of position gradient

$$\frac{\partial u(\mathbf{r},t)}{\partial t} = \nabla \cdot [d(|\nabla u(\mathbf{r},t)|)\nabla u(\mathbf{r},t)], \qquad u(\mathbf{r},0) = I(\mathbf{r}), \tag{7}$$

where $I(\mathbf{r})$ is the original image and $d(|\nabla u|)$ is a generalized diffusion coefficient which is so designed that its values are very small at the edge of an image. Perona and Malik suggested the Gaussian $d(x) = \exp[-x^2/2\sigma^2]$ and the Lorentz $d(x) = [1 + (x/\sigma)^2]^{-1}$, where $\sigma$ is a constant to be tuned for a particular application. The Perona-Malik equation has recently stimulated much interest in image processing and applied mathematical communities [5, 10–19]. It is commonly believed that the Perona-Malik equation facilitates a new and potentially more effective algorithm for noise removing, image restoration, edge detection, and image enhancement.

Catté et al. [11] pointed out the possibility of generating additional maximum and minimum which do not belong to the initial image data. The problem was analyzed in detail by Kichenassamy [12]. Further studies [5, 11, 13, 19] showed that the anisotropic diffusion process may break down when the gradient generated by noise is comparable to image edges and features. A preconvolution with a smoothing function, such as a Gaussian filter was proposed as a regularization procedure to alleviate the instability. Such a preprocess in fact degrades image quality. One of the present authors [20] proposed a statistical method which discriminates noise from image edges. The idea is to choose $\sigma$ in the Gaussian or Lorentz as a local statistical variance of $|\nabla u|$,

$$\sigma(\mathbf{r},t) = \overline{|\nabla u - \overline{\nabla u}|^2}, \tag{8}$$

where $\overline{X(\mathbf{r})}$ denotes the local average of $X(\mathbf{r})$ centered at point $\mathbf{r}$. The area of the local average can be selected in particular application. This simple approach works well in restoration of noisy images. More sophisticated approaches, including variational methods, curvature, and active contours, have been extensively explored in the literature [21–26].

Another problem with the original Perona-Malik equation concerns its efficiency in noisy removing and image enhancement. This problem was addressed by Wei [20] by introducing high-order edge-controlled diffusion operators. A fourth-order variation-based PDE was proposed by Chan et al. [27] for image restoration. You and Kaveh [28] introduced a fourth-order curvature diminishing diffusion equation. The high-order Perona-Malik equation [20] takes the form

$$\frac{\partial u(\mathbf{r},t)}{\partial t} = \sum_q \nabla \cdot \left[d_q(u(\mathbf{r},t), |\nabla u(\mathbf{r},t)|)\nabla\nabla^{2q}u(\mathbf{r},t)\right] + e(u(\mathbf{r},t), |\nabla u(\mathbf{r},t)|). \tag{9}$$

Solute-solvent dielectric interfaces here $d_q(u,|\nabla u|)$ are edge-sensitive diffusion functions and $e(u(\mathbf{r},t),|\nabla u(\mathbf{r},t)|)$ is an edge-sensitive (kinetic) production term. Numerical experiments were carried out in [20] for a simplified version of (9):

$$\begin{aligned}\frac{\partial u(\mathbf{r},t)}{\partial t} &= \nabla \cdot [d_1(u(\mathbf{r},t), |\nabla u(\mathbf{r},t)|)\nabla u(\mathbf{r},t)] \\ &\quad + \nabla \cdot [d_2(u(\mathbf{r},t), |\nabla u(\mathbf{r},t)|)\nabla\nabla^2 u(\mathbf{r},t)] \\ &\quad + e(u(\mathbf{r},t), |\nabla u(\mathbf{r},t)|).\end{aligned} \tag{10}$$

A linear and discrete version of the fourth-order diffusion operator was tested for time series analysis [6]. Clearly, the motivations behind the generalized Perona-Malik equation (10) include high-order phenomenological kinetic equations for pattern formation in alloys, glasses, polymer, combustion, and biological systems, and hyperdiffusion used to stabilize the direct numerical simulation of turbulence flow by damping small-scale oscillations around the Nyquist frequency. Image sharpening can be achieved by a balance between forward diffusion and backward diffusion in (10) via appropriately choosing the signs of $d_1$ and $d_2$. Bertozzi and Greer [29–31] have carried out mathematical analysis of the generalized Perona-Malik equation (10). The latter was found to be efficient for treating medical magnetic resonance images [32]. Gilboa et al. [33] constructed an efficient image sharpening scheme by using (10) with a triple-well potential for the kinetic production $e(u(\mathbf{r},t),|\nabla u(\mathbf{r},t)|)$. Witelski and Bowen [34] proposed alternating-direction implicit (ADI) schemes for the integration of (9).

The other problem in the Perona-Malik equation is its inefficiency for image edge detection, particularly for edge detection of images with a large amount of texture. This is due to the fact that edge detection is naturally a highpass filtering operation, while the diffusion process is inherently a lowpass filtering process. Wei and Jia [35] address this problem by introducing a pair of weakly coupled nonlinear equations:

$$u_t = F_1(u, \nabla u, \nabla^2 u, \ldots) + \varepsilon_1(v - u), \tag{11}$$

$$v_t = F_2(v, \nabla v, \nabla^2 v, \ldots) + \varepsilon_2(u - v), \tag{12}$$

where $\varepsilon_1$ and $\varepsilon_2$ are the coupling strengths. Here, $F_1$ and $F_2$ are nonlinear functions and can both be chosen as the Perona-Malik operator

$$F_q = \nabla \cdot [d_q(|\nabla u|)\nabla u], \quad q = 1, 2, \tag{13}$$

but evolve at entirely different time scales. For complicated application, the generalized Perona-Malik operator (10) can be used. This approach was motivated by the synchronization analysis of two nonlinear chaotic systems. Image edges are detected by the so-called synchronization residual at an appropriate time:

$$R(\mathbf{r}, t) = u(\mathbf{r}, t) - v(\mathbf{r}, t) \tag{14}$$

provided that the common initial value is a digital image field $u(\mathbf{r}, 0) = v(\mathbf{r}, 0) = I(\mathbf{r})$. This coupled PDE approach has been shown [35] to be very robust and efficient, and it provides superior results in image edge detection compared to those obtained by using other existing approaches, such as the Sobel, Prewitt, and Canny operators, and by anisotropic diffusion.

Furthermore, the time in the evolution equation is an extra parameter for images and often does not have much justification for its necessity. In particular, there is no rigorous and common rule to determine the period of the time evolution. An automatically controlled generalized Perona-Malik operator was proposed [20] by introducing a time-dependent factor

$$\frac{e_0}{t + t_0}, \tag{15}$$

where $e_0$ and $t_0$ are positive constants. Since each term in the generalized Perona-Malik equation may have a different time dependence, this actually turns the time into an additional dimension for optimizing image properties. Such an idea was adopted and the related technique was further explored by Gilboa et al. [36]. They proposed two time-dependent diffusion coefficients for (7) in the form of

$$d(|\nabla u|, t) = \left[1 + \left(\frac{|\nabla u|}{k(t)}\right)^2\right]^{-1}, \tag{16}$$

with $k(t) = 1/(\epsilon + \alpha_1 t)$ and

$$d(|\nabla u|, t) = \left[1 + \left(\frac{|\nabla u|}{k}\right)^{\alpha_2 t}\right]^{-1}, \tag{17}$$

where $\alpha_1, \alpha_2$ are parameters for controlling the diffusion rate, $\epsilon$ is a constant, and $k$ is the gradient threshold. Controlled evolution is an important concept for Perona-Malik type of evolution equations. In fact, two types of controls have been introduced in the literature. One control, originally proposed by Wei [20], is to control the magnitude of an individual operator as an explicit function of time, such as (15), so that its effect on the image can be optimized. The other is to control the exit time of the time evolution based on the characteristic of the evolving image. The second type of control was proposed by Wei and Jia [35] to stop the time evolution of their couple edge detection equations based on the difference in variance of two evolving images. Certainly, more research work on the automatic control of time evolution is required in order to make PDE-based image processing a really competitive approach against a host of other methods, such as wavelets, Wiener filter, neural networks, and so forth.

One advantage of PDE-based image processing methods is being elegant and mathematically rigorous. Another advantage is their readiness for systematic generalization to the processing of three-dimensional (3D) and higher-dimensional data. Geometric selectivity and parameter controllability also endow these methods great flexibility and potential for a variety of applications in medical imaging, astronomic imaging, pattern recognition, and computer vision. Nevertheless, there are obstacles which prevent PDE-based image processing methods from being used in practical applications, that is, industrial codes. First of all, multiple iterations involved in solving nonlinear PDEs are in general much more expensive than the single-step operation of most classic image processing methods. This lack of efficiency is very severe for the processing of large 3D medical image data. Moreover, the numerical solution of Perona-Malik type of nonlinear PDEs is nontrivial. Numerical instability exists when the time step is too large in explicit schemes. For implicit schemes, stability may not be a problem, but other undesired features might be introduced to the solution.

Many efficient solution techniques were proposed [37–40] for the linear heat equation. However, when the emphasis of the diffusion process is changed from the linear filtering to the nonlinear one, poor computational efficiency becomes a main obstruction in the practical applications of the nonlinear diffusion equation. In time, explicit schemes like forward Euler (first-order), multilevel explicit finite-difference schemes are most widely used. However, these explicit schemes require very small time steps in order to be stable. It is doomed that the whole diffusion procedure is rather time consuming. Consequently, accelerated explicit schemes are desirable. A fast explicit numerical scheme ($\lambda$-resolution) was presented to approximate the solution of the linear diffusion filtering [41]. More sophisticated and absolutely stable approaches are semi-implicit schemes [11, 18]. Weickert et al. [18] proposed an additive operator splitting (AOS) scheme for nonlinear diffusion process. In contrast to traditional multiplicative splitting such as the ADI and locally one-dimensional splitting, all axes are treated in the same manner in AOS scheme. However, this scheme needs to compute matrix inversions which are usually a source of computational errors and require much computer memory. In space, since the pixel structure of digital images provides a natural discretization on a fixed rectangular grid, it is not surprising that finite-difference-based methods, such as second-order, fourth-order, and sixth-order central finite-difference schemes, are commonly used for the task of discretization. Alternatives to the finite-difference methods include finite-element methods [42–44], finite-volume schemes [45, 46], pseudospectral approaches [47], wavelets [20, 47, 48], multigrid methods [49], fast level-set methods [50], high-order ENO [51], lattice Boltzmann methods [52], and stochastic simulations [53].

Recently, a local spectral (LS) method, also called the discrete singular convolution (DSC) [54], was proposed as an efficient numerical tool for solving PDEs. The mathematical foundations of the DSC algorithm are the theory of distribution and wavelet analysis. The DSC algorithm has found to be useful in a number of scientific and engineering applications [55–63]. More recently, a family of local spectral evolution kernels (LSEKs) [64] have been constructed for analytically integrating a class of evolution PDEs:

$$\frac{\partial}{\partial t} f(x,t) = \left(A(t)\frac{\partial^2}{\partial x^2} + B(t)\frac{\partial}{\partial x} + C(t)\right) f(x,t), \tag{18}$$

where $A$, $B$, and $C$ are functions of time, and $\text{Re}(A) \geq 0$. The proposed local spectral method has the same level of accuracy as that of spectral methods in space, and is analytic in time. The local spectral evolution kernels are constructed by using Hermite functions. However, unlike the standard global Hermite spectral methods [65], the LSEKs adopt uniform grids and have banded matrices, just like other DSC kernels. In fact, the length of the computational stencil, and thus the accuracy of the LSEK, can be controlled according to the needs of the problem of interest, which is another feature of the DSC inherited from wavelet analysis. Moreover, the local property endows the LSEK method with sufficient flexibility to handle moderately complex geometry and complex boundary conditions. In principle, Fourier spectral methods can also solve many evolution equations analytically. However, they are restricted to periodic boundary conditions and lead to distortion near image boundaries.

The objective of the present paper is to introduce an evolution-operator-based single-step image processing method. The LSEK systematically incorporates anisotropic diffusion coefficients at each pixel. The new method arrives at a desired evolution time in a single step. Moreover, the proposed method is free of instability constraint and is of spectral accuracy for integrating evolution PDEs (18). Furthermore, by an appropriate design of coefficient $C(t)$, one can achieve the automatic termination of the time integration. In this paper, we explore the use of the proposed single-step method for several important tasks, including image deblurring, denoising, edge detection, and enhancement. Image denoising is accomplished by incorporating anisotropic diffusion type of coefficients in the LSEK, while image deblurring is realized by an appropriate forward-backward diffusion process. The coupled PDE system (11) is simplified and utilized as a model for constructing new schemes for image edge detection, and the resulting image edge is employed for image enhancement. It is believed that the proposed evolution-operator-based method is one of the fastest schemes for image processing. In particular, the proposed PDE-based method has great potential for processing large-scale multidimensional data and for data mining.

This paper is organized as follows. In Section 2, the theory of local spectral evolution kernel (LSEK) is briefly reviewed. The frequency response of the LSEK is studied. The efficiency of the LSEK is examined by 1D and 2D diffusion processes that are exactly solvable. Numerical results are compared with those obtained by using other standard methods. A LSEK-based quasinonlinear method is proposed for image processing. Section 3 is devoted to the applications of the proposed method. Computer experiments are conducted to demonstrate the performance and efficiency of the proposed single-step method for a number of image processing tasks. This paper ends with a conclusion.

### 2. THEORY AND ALGORITHM

In this section, we give a brief review on the local spectral method and its evolution kernels. Discrete Fourier analysis is carried out to study the filter properties of the LSEK. Accuracy of the the kernel and efficiency of the scheme are examined. A new evolution-operator-based method is introduced for image processing.

### 2.1. Local spectral evolution kernels

The discrete singular convolution (DSC) is a general framework for constructing local spectral methods. It is an efficient approach for the numerical realization of singular convolution

$$F(t) = (T * \eta)(t) = \int_{-\infty}^{\infty} T(t-x)\eta(x)dx, \tag{19}$$

where $\eta(x)$ is an element of the space of test functions, and $T(t-x)$ a singular kernel. Interesting examples include singular kernels of delta type and Hilbert (Able) type. The latter plays an important role in the theory of analytic functions, processing of analytical signals, theory of linear responses, and Radon transform. The delta-type kernels are of the form

$$T(x) = \delta^{(n)}(x), \quad n = 0, 1, 2, \dots, \tag{20}$$

where $\delta(x)$ is the delta distribution. We focus on these singular kernels since they are key elements in the approximation theory and the numerical solution of differential equations. Because of their singular nature, their approximation is necessary for numerical computations. A variety of candidates are available for the approximation in the literature. Among these examples, Shannon's delta kernel is of particular interest. It essentially gives rise to classic Fourier spectral methods. Some of local spectral kernels [54] are constructed by the regularized Shannon kernel

$$\delta_{h,\sigma}^{(l)}(x-x_k) = \frac{d^l}{dx^l}\frac{\sin(\pi/h)(x-x_k)}{(\pi/h)(x-x_k)}\exp\left[\frac{-(x-x_k)^2}{2\sigma^2}\right], \tag{21}$$

where $h$ is the grid spacing. The delta distribution can also be approximated by classical polynomials. Korevaar [66] proposed a Hermite function approximation to the delta distribution. Independently, Hoffman et al. [67] derived the same approximation and an expression for its arbitrary derivatives

$$\delta_{h,\sigma}^{(l)}(x-x_k) = \frac{(-1)^l h}{2^{l/2}\sigma^{l+1}}\sum_{n=0}^{M_h}\left(-\frac{1}{4}\right)^n \frac{1}{\sqrt{2\pi}n!}h_{2n+l}\left(\frac{x-x_k}{\sqrt{2}\sigma}\right), \quad l = 0, 1, 2, \dots, \tag{22}$$

where the Hermite function $h_n$ is defined as

$$h_n(x) = \exp(-x^2)H_n(x). \tag{23}$$

Here $H_n(x)$ is the classic Hermite polynomial.

In the DSC algorithm, a function and its derivatives can be approximated by

$$g^{(l)}(x) = \sum_{k=-M}^{M} \delta_{h,\sigma}^{(l)}(x - x_k)g(x_k), \quad l = 0, 1, 2, \ldots, \tag{24}$$

where $2M + 1$ is the computational bandwidth, or effective kernel support. This approach has been extensively validated by solving nonlinear PDEs [58, 61], vibration analysis [59, 60], Maxwell's equations [55, 57], incompressible and compressible Navier-Stokes equations [62, 63], and image edge detection [56]. It is also used in this work for computing derivatives.

What is crucial for the present work is an analytical integrator of the PDEs given in (18). Their formal solution can be expressed as

$$f(x,t) = \int dx' K(x,x',t,t')f(x',t'), \tag{25}$$

where $K(x,x',t,t')$ is an evolution operator, defined as

$$K(x,x',t,t') = \left(x, \exp\left(\int_{t'}^{t} L\,dt\right)x'\right). \tag{26}$$

Here the standard inner product $(f,g)$ is defined as $(f,g) = \int f^*(x)g(x)dx$. The operator can be converted into an algebraic expression by using a Fourier projection operator and then carrying out the integration over the wavenumber. The resulting kernel is diagonal in the position representation $K(x,x',t,t') = K(x-x',t,t')$ and can be cast in a local spectral representation

$$K_{h,\sigma}(x,t,t') = \frac{1}{h}\int K(x-x',t,t')\delta_{h,\sigma}^{(0)}(x')dx'. \tag{27}$$

One therefore obtains [64]

$$K_{h,\sigma}(x,t,t') = \frac{h}{\sigma}e^{C_{t'}^{t}} \sum_{n=0}^{M_h/2}\left(-\frac{1}{4}\right)^{n} \frac{1}{\sqrt{2\pi}n!}\left(\frac{\sigma}{\sigma_{t'}^{t}}\right)^{2n+1} h_{2n}\left(\frac{x+B_{t'}^{t}}{\sqrt{2}\sigma_{t'}^{t}}\right), \tag{28}$$

where $X_{t'}^{t} = \int_{t'}^{t} X(s)ds$, for $X = A, B$, and $C$. Here $\sigma_{t'}^{t}$ is given by

$$\sigma_{t'}^{t} = \sqrt{\sigma^2 + 2A_{t'}^{t}}. \tag{29}$$

Expression (28) is called a local spectral evolution kernel (LSEK). The case of $A = 0$ can be recovered by setting $\sigma_{t'}^{t} = \sigma$. Another simplification occurs when $A, B$, and $C$ are independent of time, that is, $X(t) = X$. Then, $X_{t'}^{t} = X(t-t') = X\Delta t$, and $K_{h,\sigma}(x,t,t') = K_{h,\sigma}(x,t-t') = K_{h,\sigma}(x,\Delta t)$.

For an initial function $f(x,t')$, the solution $f(x,t)$ of (18) at an arbitrary time $t$ can be analytically given by

$$f(x,t) = \sum_{k=-M}^{M} K_{h,\sigma}(x-x_k,t,t')f(x_k,t'), \tag{30}$$

or for a grid point $x_j$,

$$f(x_j,t) = \sum_{k=-M}^{M} K_{h,\sigma}(kh,t,t')f(x_j-kh,t'). \tag{31}$$

This implementation is the same as that for other local spectral kernels, given in (24). Equation (31), together with (28), implies that the evolution operation has been converted into an algebraic operation and the local spectral solution (31) to PDEs given in (18) is analytical. This approach is therefore free of stability constraint and is in principle of spectral accuracy when the support of the stencil is sufficiently large.

For image and data analysis, it is necessary to deal with higher dimensions. If the operator in (18) is of $q$-dimensional,

$$\begin{aligned}\frac{\partial}{\partial t}&f(x1,\ldots,xi,\ldots,xq,t)\\ &= \prod_{xi=x1}^{xq}\left[A_i(t)\frac{\partial^2}{\partial xi^2} + B_i(t)\frac{\partial}{\partial xi} + C_i(t)\right]\\ &\quad\times f(x1,\ldots,xi,\ldots,xq,t),\end{aligned} \tag{32}$$

its solution on an arbitrary grid point $(x1_{j1},\ldots,xi_{ji},\ldots, xq_{jq},t)$ can easily be constructed by tensor product

$$\begin{aligned}f&(x1_{j1},\ldots,xi_{ji},\ldots,xq_{jq},t)\\ &= \prod_{xi=x1}^{xq}\left[\sum_{k_{xi}=-M_i}^{M_i} K_{h_i,\sigma_i}(k_{xi}h_i,t,t')\right]\\ &\quad\times f(x1_{j1}-k_{x1}h_1,\ldots,xi_{ji}-k_{xi}h_i,\ldots,xq_{jq}-k_{xq}h_q,t'),\end{aligned} \tag{33}$$

where definitions of quantities with subscripts are self-evident.

The local spectral method also provides a freedom to choose a desired level of accuracy so that the computing time is reduced. This can be achieved by an appropriate choice of $M_h$, $\sigma$, and $M$. Both forward and backward time evolutions can be resolved by using the LSEK as long as $\mathrm{Re}(\sigma_{t'}^{t}) \geq 0$ for a given $t$. A detailed analysis and validation of the LSEK including an efficient algorithm for solving complex nonlinear PDEs via time splitting formulation are out of the scope of the present work and have been accounted elsewhere [64].

### *2.2. Filter properties*

To achieve a better understanding of the LSEK, we study its filter properties by discrete Fourier analysis. Such a study allows us to optimally choose the DSC parameters, that is, the computational bandwidth $M_h$ and the scaled Gaussian window size $r = \sigma/h$. The Fourier analysis is a classical technique for characterizing the Fourier resolution of an interpolation or differentiation scheme applied to a class of compactly supported periodic functions and for examining the frequency response of a filter.

Note that if the function $f(x,t)$ in (30) is regarded as a two-parameter family of signal, the convolution kernel

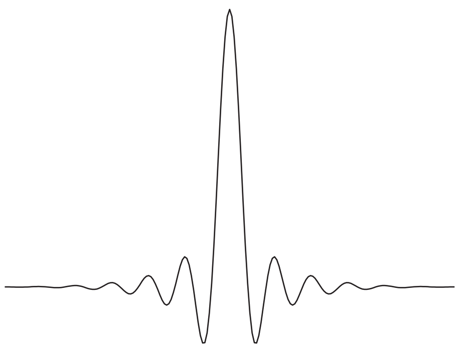

Figure 1: The LSEK in the coordinate domain with $A(t) = B(t) = C(t) = 0$.

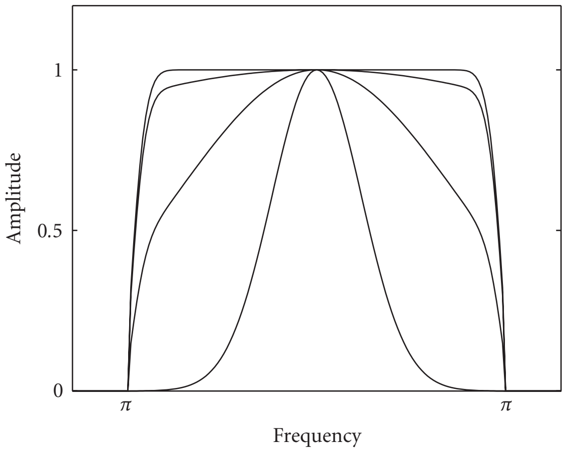

Figure 2: The frequency responses of the LSEK with $B(t) = C(t) = 0$, $M = 64$, and $M_h = 88$. The solid lines from outside to inner are for $A_{t'}^{t} = 0, 0.001\sigma^2, 0.01\sigma^2$, and $0.1\sigma^2$.

$K_{h,\sigma}(x - x_k, t, t')$ is a linear time-invariant system and is practically used as a finite impulse response (FIR) filter. This filter is stable and inevitable for $C < \infty$. On the one hand, when $B = C = 0$ and $A(t)$ is a constant, (18) reduces to the heat equation. As the LESK provides analytical solution to the heat equation, one might assume that the analytic integrator is a Gaussian filter as the action of heat equation is known to be equivalent to the Gaussian filter [2]. However, this is not true. In fact, it looks like a wavelet filter in the coordinate domain, see Figure 1. Mathematically, it is called a kernel of Dirichlet type in contrast to the kernel of positive type, for example, a Gaussian kernel. On the other hand, when $B = 0$, the kernel $K_{h,\sigma}(x, t, t')$ is obviously symmetric and its Fourier response is of linear phase and real, since it contains only even terms of the Hermite functions. As it is also the evolution kernel for the parabolic PDE (when $A \neq 0$), it must be a lowpass filter in general for $t > t'$ as shown Figure 2. It is noted that the LSEK lowpass is almost ideal for small $t$, and appropriate $M_h$ and $r$ values. In general, large $M_h$ and $r$ values lead to a better approximation to the ideal lowpass filter. Nevertheless, since they are constructed from polynomial

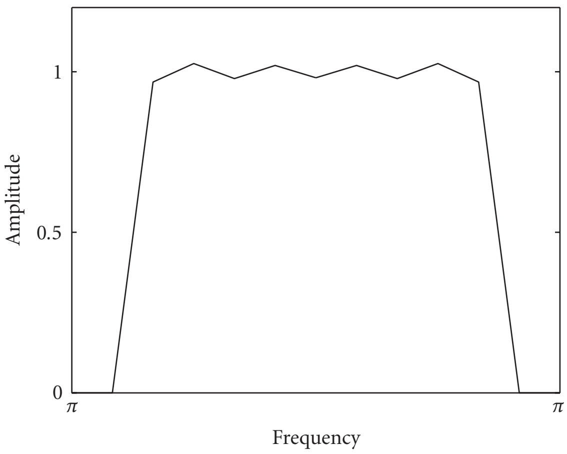

Figure 3: The frequency response of the LSEK with $A(t) = B(t) = C(t) = 0$, $M = 6$, and $M_h = 88$.

approximation, they are Chebyshev type of filters as illustrated with appropriate $M_h$ and $r$ values, see Figure 3. Moreover, for a given set of other parameters, when $t - t'$ is getting larger, that is, long time evolution, the LSEK filter response becomes more and more focused in the low-frequency part, corresponding to large amount of diffusion.

In general, the parameter $B$ gives rise to a translation in the coordinate, which leads to an extra phase factor in the frequency response, see Figure 4. Obviously, in signal analysis, it is the time-shift factor that is useful for the circuit design. In other words, the drafting term $B(t)(\partial/\partial x)$ in (18) is a time-shift operator from the point view of signal processing. Since $B(t)$ is a function of time, it in fact can be used for nonlinear time shifting. As shown in Figure 5, the parameter $C$ provides an overall scaling factor (either exponential growth or decay) to the amplitude of the signal, which can be useful in practical applications.

### 2.3. Numerical test

We illustrate the proposed local spectral method for the diffusion equation in this section. The diffusion equation is a special case of the class of PDEs of (18) ($B = 0$ and $C = 0$). The most important fact is that the diffusion equation has extensive applications in image processing. The accuracy of the proposed local spectral method is obtained by comparing its results with exact solutions. Two advantages of the present local spectral approach (accurate and fast) have been fully demonstrated since both forward and backward time evolutions can be resolved by using the LSEK as long as $\mathrm{Re}(\sigma_{t'}^{t}) \geq 0$ for a given $t$. When $A_{t'}^{t}$ is positive in (29), it is obviously a forward propagation process. In this case, the numerical solution of the forward diffusion equation at any time can be obtained by (31) in only one time step. However, when time $A_{t'}^{t}$ is negative, the problem becomes a backward propagating process, and we still can use (31) to get the solution as long as we ensure $\mathrm{Re}(\sigma_{t'}^{t}) \geq 0$. However, the backward diffusion is an unsteady problem. In order to obtain a reliable numerical solution, we have to terminate the backward diffusion

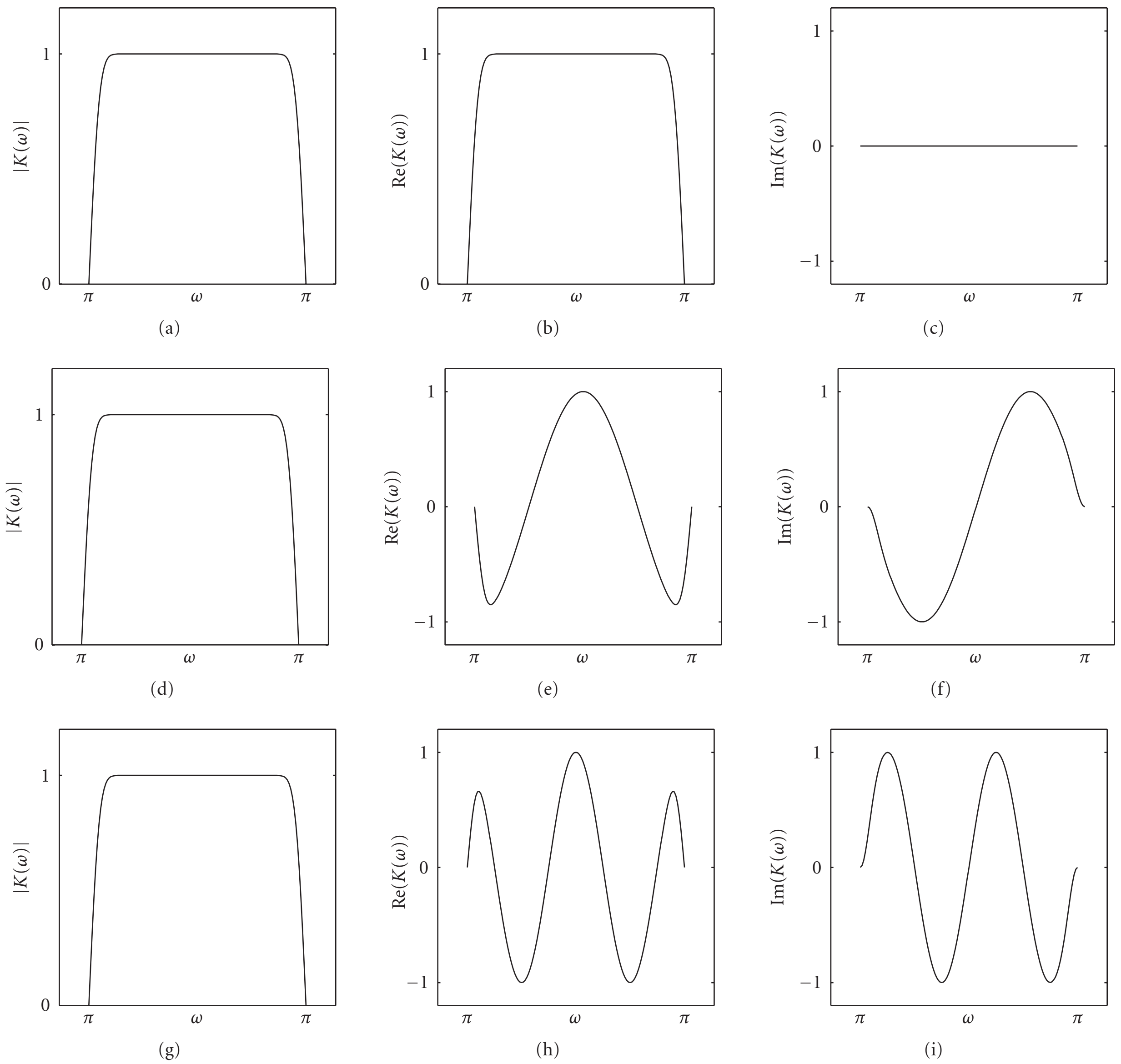

Figure 4: The frequency responses of the LSEK with $M = 64$, $M_h = 88$, $A(t) = C(t) = 0$, and different $B^t_{t'}$: (a)–(c) $B^t_{t'} = 0$; (d)–(f) $B^t_{t'} = h$; (g)–(i) $B^t_{t'} = 2h$.

in a limited time. As was mentioned earlier, the accuracy of the proposed local spectral method is controllable by adjusting its support parameter $M$ and the Hermite parameters $M_h$ and $\sigma$. In this numerical example, we choose the Hermite parameters and the computational bandwidth as $M_h = 128$, $\sigma = 3.7\Delta$, and $M = 45$ to illustrate the spectral order of accuracy of the method. Unless stated otherwise, the above set of parameters is used in this section. In Example 2 (2D diffusion process), we will compare the performance of the present local spectral method with that of Fourier spectral method which is realized by using the fast Fourier transformation (FFT).

*Example 1.* We consider a one-dimensional linear diffusion process given by

$$\frac{\partial f(x,t)}{\partial t} = \alpha \frac{\partial^2 f(x,t)}{\partial x^2}, \tag{34}$$

where $\alpha$ is the diffusion coefficient. With an initial delta-function distribution localized at $x_0$, the analytic solution is

$$f(x,t) = \sqrt{\frac{1}{4\alpha\pi(t-t_0)}}\, e^{-(x-x_0)^2/[4\alpha(t-t_0)]}. \tag{35}$$

The computation is conducted using a sufficiently large interval $[-10, 10]$ of coordinate space to ensure that boundary reflection is negligible. Fifty grid points are used for this interval. The diffusion coefficient $\alpha$ is set to be 0.5 in all computations. Since it is difficult to accurately represent a delta function on a grid as the initial condition, we choose the initial value as the Gaussian of (34) at $t = 1.0$ for the forward diffusion process and the Gaussian of (34) at $t = 2.0$

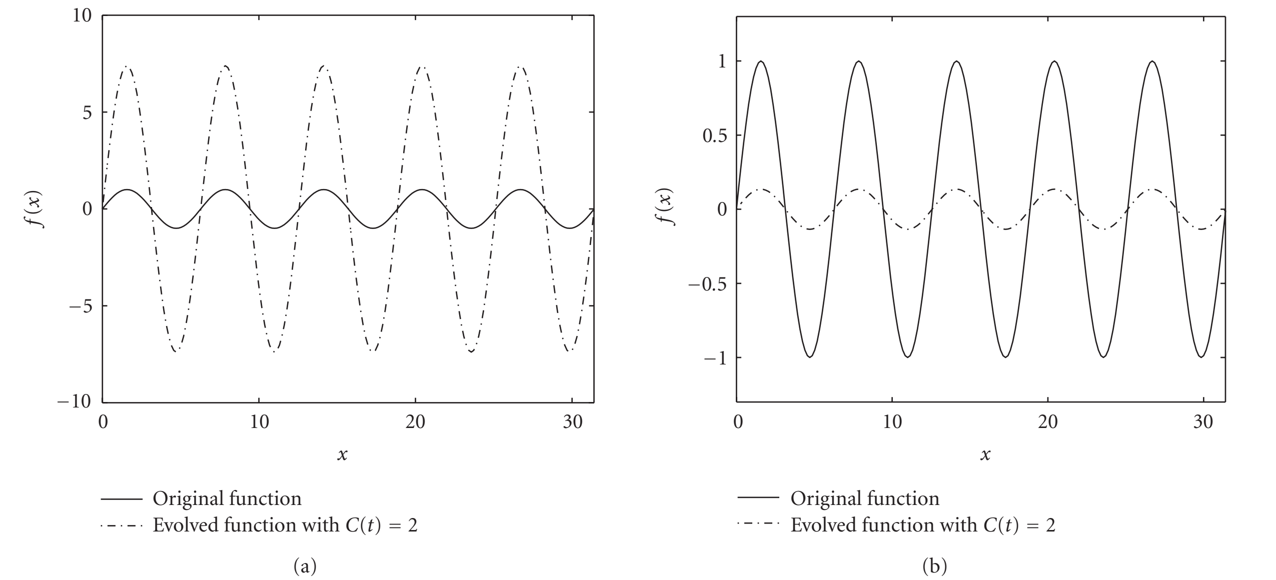

Figure 5: The frequency responses of the LSEK with $M = 64$, $M_h = 88$, $A(t) = B(t) = 0$, and different $C_{t'}^t$: (a) $C_{t'}^t = 2$; (b) $C_{t'}^t = -2$.

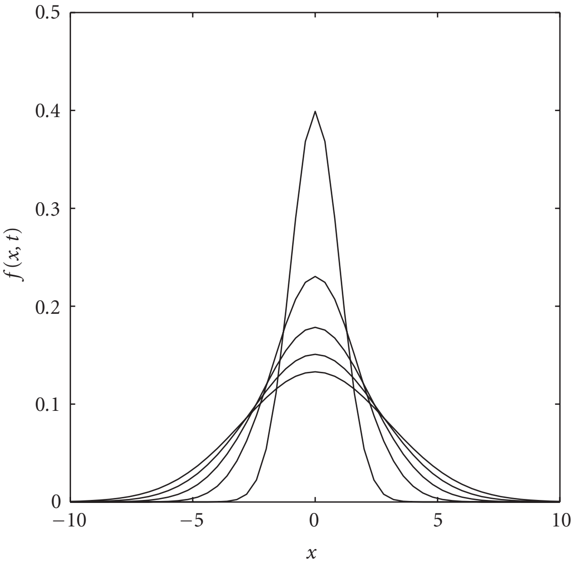

Figure 6: The numerical solutions of the 1D diffusion equation. The centerlines in the descending order are at time $t = 1.0, 3.0, 5.0, 7.0$, and 9.0.

as the initial condition for the backward diffusion process. The numerical solutions at time $t = 1.0, 3.0, 5.0, 7.0$, and 9.0 are given in Figure 6. Two kinds of numerical error measures, that is, $L_\infty$ and $L_2$ are used to evaluate the quality of the present local spectral method. The computational errors are listed in Table 1. It can be observed from Table 1 that the present local spectral method gives highly accurate numerical results. On the other hand, it can be seen clearly that the results of the backward diffusion are not as good as those of the forward diffusion. The loss of accuracy is due to the fact that the backward diffusion is itself an unsteady process. Moreover, since no time evolution scheme is needed in the present method, there is no accumulation of time discretization error. This property guarantees the accuracy and the speed of the present method and shows great advantage over many other numerical algorithms.

*Example 2.* We consider a two-dimensional linear diffusion equation

$$\frac{\partial I(x,y,t)}{\partial t} = a^2\left[\frac{\partial^2 I(x,y,t)}{\partial x^2} + \frac{\partial^2 I(x,y,t)}{\partial y^2}\right]. \quad (36)$$

It is a good test problem because it admits an exact solution of the form

$$I(x,y,t) = \frac{1}{4a^2\pi t}e^{-(x^2+y^2)/4a^2 t}, \quad (37)$$

where $a$ is a positive parameter and we will use $a = 0.7$ in this test. To compare with the FFT method, we impose periodic boundary conditions in both $x$- and $y$-directions. The computation domain is taken to be $[0, 10] \times [0, 10]$ with equal spatial discretization. Although the grid can be set to be any integer values for the LSEK method, for the purpose of comparing with the FFT method in which the grid is required to be the power of 2, we choose $N_x = N_y = 32$ in our computation. The forward diffusion is initialized to be a Gaussian wave at $t = 0.1$,

$$I_0(x,y) = \frac{1}{4a^2\pi t}e^{-[(x-x_0)^2+(y-y_0)^2]/4a^2 t}, \quad (38)$$

where the point $(x_0, y_0)$ is the center of the 2D square domain. The detailed LSEK method used for the 2D problem is given in (33). Our interest is to explore the efficiency of the present LS method. Therefore, we will not only compare the numerical errors of the LSEK method and the FFT method but also the computing time of both methods. They are given in Table 2. It is clear from Table 2 that the present LSEK method is as accurate and fast as the FFT method when they are solving the diffusion equations.

By comparing the present local spectral method and the Fourier spectral method, we reach conclusions which are summarized as follows.

(i) The accuracy of the proposed LSEK method is controlled by its computational bandwidth $M$, Hermite parameters $M_h$ and $\sigma$. Under choice of $M=45$, $M_h$=128, and $\sigma$=3.7$\Delta$, the LS method can provide the same level of accuracy as that of Fourier spectral method.

(ii) The global nature of the Fourier spectral method makes it difficult for practical applications to problems of complicated boundary condition and complex geometry. However, the present LSEK is a local method which can be used for treating complicated boundary conditions and geometries.

(iii) For the Fourier spectral method, the number of grid points is required to be the power of 2, but there is no such a limitation for our LS method.

(iv) The present LSEK method transforms the diffusion equation into algebraic calculations. This implies that the LSEK method can speed up the computation dramatically, and thereby reduce the computational cost. Specifically, the CPU cost of the LSEK scales as $O(MN)$, where $M$ and $N$ are half of computational bandwidth and the number of grid points to deal with. However, asymptotically, the LSEK method requires less CPU time than the Fourier pseudospectral method does as the latter scales as $O(N\log N)$.

In practical applications, especially in image processing, the aforementioned precision is entirely unnecessary. A three-percent error does not produce much difference in visual perception. Therefore, in the following image experiments, we choose $M_h = 36$, $\sigma = 1.7\Delta$, and $M = 8$. In fact, even less costing parameters can be used for certain tasks in image processing, such as image deblurring and denoising where the image quality is very low to start with.

### 2.4. Adaptation of anisotropy

Obviously, the Perona-Malik type of anisotropic diffusion equations cannot be solved directly with the LSEK in a single step, albeit the local spectral method given in (24) can be used to solve these equations iteratively. Fortunately, a close examination on the solution scheme, (31) or (33), and the LSEK (28), one realizes that the solution scheme is of collocation type and the kernel allows a localized modification of coefficients $A, B$, and $C$, that is,

$$A \longrightarrow A(|\nabla I|), \qquad B \longrightarrow B(|\nabla I|), \qquad C \longrightarrow C(|\nabla I|). \tag{39}$$

This flexibility of selecting local coefficients endows us with a new single-step evolution-operator-based method for image processing and data analysis.

In Perona-Malik equation (7), the break up of the operator leads to a gradient controlled diffusion term and a gradient controlled drafting term. The local strength of the both terms can be easily computed and incorporated in the LSEK expression.

Table 1: Errors of the solutions for the 1D diffusion equation.

| Forward diffusion | | | Backward diffusion | | |
|---|---|---|---|---|---|
| Time | $L_2$ | $L_\infty$ | Time | $L_2$ | $L_\infty$ |
| 2.0 | 7.17E-17 | 2.22E-16 | 1.8 | 8.25E-13 | 6.50E-13 |
| 3.0 | 3.72E-17 | 8.33E-17 | 1.6 | 2.32E-11 | 1.01E-10 |
| 4.0 | 3.80E-17 | 1.39E-16 | 1.4 | 1.19E-09 | 4.28E-09 |
| 5.0 | 9.53E-16 | 6.94E-17 | 1.2 | 1.74E-07 | 5.90E-07 |

Table 2: Errors of the solutions and the CPU time for the 2D diffusion equation.

| | FFT | | | LSEK | | |
|---|---|---|---|---|---|---|
| Time | $L_2$ | $L_\infty$ | CPU time | $L_2$ | $L_\infty$ | CPU time |
| 0.5 | 1.31E-7 | 2.16E-7 | 0.0156 | 1.08E-7 | 1.70E-7 | 0.0156 |
| 1.0 | 5.10E-7 | 4.69E-7 | 0.0156 | 7.08E-7 | 4.69E-7 | 0.0156 |
| 1.5 | 2.84E-5 | 2.20E-5 | 0.0156 | 3.81E-5 | 2.20E-5 | 0.0156 |
| 2.0 | 2.08E-4 | 1.38E-4 | 0.0156 | 2.70E-4 | 1.38E-4 | 0.0156 |

Since the diffusion coefficients computed from Perona-Malik type of equations may vary from point to point, possible $N^2$ sets of LSEK weights may be computed. This requires much computation if $N$ is about 512. On the other hand, since image resolution is normally limited to 8 bits, it is obviously unnecessary to use more than 256 sets of LSEK weights. We therefore classify the diffusion coefficients into a total of $N_c$ groups. For each group, we calculate a set of LSEK weights according to the mean value of the group. In most practical applications, taking $N_c \sim 20$ is enough and it costs less than 1 second to compute these weights. In the above argument, we have assumed that the drafting coefficient behaves similar to the diffusion coefficient at each point. If this is not the case, the discussed classification method can be easily modified. Thus, the basic procedure for the proposed evolution-operator-based image processing method is the following.

(i) Compute local coefficients $A(|\nabla I|)$, $B(|\nabla I|)$ and $C(|\nabla I|)$ from an image function $I(x, y)$ and classify the weights into a total of $N_c$ groups.

(ii) For each given group of diffusion coefficients, the group's mean value is taken as the diffusion coefficient, labeled as $A_c$ and $B_c$. Then compute a set of $M+1$ LSEK coefficients. Here we assume a uniform grid in both $x$- and $y$-directions and a common stencil size of $2M + 1$. Due to the symmetry of the LSEK, only $M + 1$ coefficients are independent.

(iii) For a pixel $I_c(x_j, y_i, t)$, whose diffusion coefficients are labeled as $A_c$ and $B_c$, its value at arbitrary time $t$ can be updated as

$$I_c(x_j, y_i, t) = \sum_{k_x=-M}^{M}\sum_{k_y=-M}^{M} K_{h,\sigma,A_c,B_c}(k_x h, t) K_{h,\sigma,A_c,B_c}(k_y h, t) \times I(x_j - k_x h, y_i - k_y h), \tag{40}$$

where $K_{h,\sigma,A_c,B_c}(k_x h, t)$ is obtained from (28) by setting $t' = 0$, $A^t_{t'} = A_c t$, and $B^t_{t'} = B_c t$.

Although the proposed method has its root in nonlinear PDEs, it does not exactly solve the anisotropic diffusion equation. Instead, it is constructed by the pointwise adaptation of anisotropy to the evolution kernel for the isotropic diffusion equation. Its major advantages are its single-step operation, freeness of numerical instability, readiness for multidimensional problems. Moreover, by appropriately choosing $B$ and $C$, systematic time shift and amplitude scaling can be easily accomplished in a single step.

### 3. APPLICATIONS

In this section, we apply the evolution-operator-based single-step approach for image deblurring, denoising, image edge detection, and image enhancement. The performance of the proposed method is illustrated by a large number of standard test images and is compared with that of other existing methods.

### *3.1. Image deblurring*

Image blur refers to serious degradation to image qualities by either the environment of an imaging object, or the process of imaging, or the processing of an image [1]. Backgrounds of imaging objects, such as fog, air pollution, texture to a fingerprint residual and illumination, are common environmental sources of image blur. Motions of the imaging object and/or camera, or out-of-focus lens generate blur in the process of imaging. Jiang and Wang [68, 69] discussed image blurring due to reconstruction techniques. Very often, image processing may lead to blur during a variety of mathematical operations, such as Gaussian convolution, interpolation, or image registration. Deblurring is a basic image processing task and the subject is so extensively studied that it is virtually impossible to give a comprehensive review to include all important contributions and developments in the literature.

Essentially, deblurring methods can be classified as linear and nonlinear, global or local, time-dependent or time-independent, a priori knowledge based or blind, and so forth. Linear filters [70–73] do not make use of local information of an image under processing, and are usually simple and low cost. Many linear filers, such as Wiener filter, Hanning filter, and Fourier filtering, work effectively when there is a priori knowledge about the source of the blur. Moreover, linear filters are often utilized to obtain initial information for other nonlinear filters. Nonlinear filters make use of the local information of an image to gain a better effect of deblurring. Some nonlinear filters assume partially a priori knowledge of an image, such as Kalman filter and Lucy-Richardson algorithm. The former makes optimal use of imprecise data on a linear (or nearly linear) system with Gaussian errors to continuously update the best estimate of system's current state, while the latter maximizes the likelihood that the resulting image is an instance of the blurred image, assuming the Poisson noise statistics. Many blind deblurring algorithms can be used effectively when no information about the source or status of the distortion (blurring and noise) is known. They utilize the local information or Fourier spectrum of an image intensively and achieve the goal of deblurring by iterations. Obviously, Perona-Malik type of PDE-based image deblurring algorithms are time-dependent, nonlinear, blind filters. Wang et al. proposed an efficient blind deblurring algorithm for spiral computed tomography (CT) images [74]. While wavelet transform is a linear algorithm in nature [75], its application to image processing can be made nonlinear by appropriately incorporating image information into the selection of truncation parameters [76–79]. Recently, some time-independent total-variation (TV-) based regularization functionals have attracted much attention because of their ability of recovering image edges during deblurring, see Rudin et al. [14], Li and Santosa [80], Dobson and Vogel [81], and Chan et al. [82]. These methods are often formulated in terms of Euler-Lagrange equations and are associated with ill-posed inverse problems. Ideally, TV-based methods should work as blind algorithms. Nevertheless, the main difficulty associated with TV methods, as indicated by many researchers, is the determination of the unknown regularization parameter(s), which balances the fidelity and smoothness of a TV solution. Consequently, these methods are most efficient when some a priori knowledge about the image is given.

A common used degradation process is often presented in terms of a blur operation and additive noise

$$I_0 = RI + \eta, \tag{41}$$

where $\eta$ stands for a white additive Gaussian noise and where $R$ is a linear operator representing the blur (usually a convolution). Given degradation version of image $I_0$, the restoration problem is then to obtain $I$ knowing (41). In this study, we will concern ourselves only with two aspects of image restoration which are deblurring and denoising.

We also make use of the sharpening property of the backward diffusion to restore a blurry image. The effect of noise amplification (an inherent byproduct of backward diffusion process) will be minimized by terminating the diffusion process in a limited time or by choosing an appropriate diffusion coefficient. We consider the anisotropic diffusion equations,

$$\begin{gathered}\frac{\partial I}{\partial t} = A(|G_\sigma * \nabla I|, t)\nabla^2 I,\\ I|_{t=0} = I_0,\\ \partial_n I|_{x\in\partial\Omega} = 0,\end{gathered} \tag{42}$$

as a model. Here $n$ is a unit vector, outward normal to the boundary $\partial\Omega$. The diffusion coefficient $A$ is a function of the smoothed gradient magnitude $s = G_\sigma * \nabla I$ evaluated at $t = 0$. We further create the LSEK analogy of (42), which is given by (40) with $B = C = 0$. Here, we design a smoothed gradient-magnitude-dependent diffusion coefficient for deblurring:

$$A(s) = \begin{cases} \dfrac{1}{\sqrt{1+(s/\epsilon)^2}} - \dfrac{0.18}{\sqrt{1+(s/k)^2}}, & s \geq 2,\\[2ex] \dfrac{1}{\sqrt{1+(2/\epsilon)^2}} - \dfrac{0.18}{\sqrt{1+(2/k)^2}}, & \text{otherwise}, \end{cases} \tag{43}$$

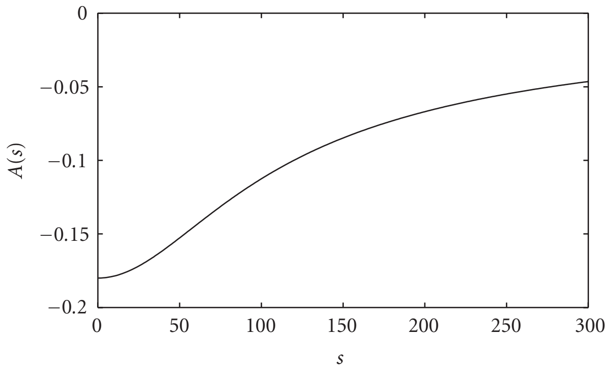

Figure 7: Diffusion coefficient $A(s)$ plotted as a function of the smoothed gradient magnitude.

where $\epsilon$ is a small strictly positive real number for avoiding zero denominator and $k$ is a threshold parameter like that in the Peronal-Malik model. Here $k$ can be a constant threshold or be adjusted locally by gradient. In the latter case, the parameter $k$ varies gradually with the image, and edge sharpening is accomplished by different thresholds in different locations. As an illustration we present in Figure 7 a plot of the suggested diffusion coefficient $A$ with $k = 80$. It is seen that the diffusion coefficient considered in this work is strictly increasing and negative. Negative diffusion coefficient is for edge sharpening and increasing tendency ensures less backward diffusion for large gradient magnitudes.

We use the parrot image for our demonstration. The blurry parrot image is obtained by convolving the initial parrot image with a Gaussian kernel ($\sigma = 2$). As for another Gaussian kernel used for the calculation of the smoothed gradient $G_\sigma * \nabla I$ of original image, we set $\sigma = 3$. We stop the diffusion process at time $t = 1.0$. Figure 8 shows the results of deblurring. For a more clear observation, we zoomed Figure 8 out near one parrot's head and the zoomed images are shown in Figure 9. We found that edges at different locations have been improved. For example, the edges near parrot's beak and those around the eye are well sharpened. Figure 10 shows the deblurring process for a building. The blurring and deblurring processes are similar to those described in the parrot image. In this case, we are interested in the features that characterize the architecture, such as edges and lines.

In fact, one can interpret the above deblurring process as an adaptive resolution of the linear diffusion equation which is conceptually different from the conventional nonlinear diffusion filtering in which the nonlinearity is spatial.

### *3.2. Image denoising*

Noise is one of the most common sources of image degradation [83]. It is often referred to as the rapid changes over spatial extension in image intensities or color plan. Either imaging acquisition process or naturally occurring phenomena can lead to noise contamination. Typically, noise is assumed to be additive and satisfies a mathematical model, such as the Poisson, or the Gaussian, or Laplacian. Some noise is multiplicative, for example, the speckle noise. The image denoising problem refers to the process of recovering an image contaminated by noise [84] and is one of the most elementary operations in image processing.

The earlier algorithms include (linear) spatial filtering approach [73] and frequency cut-off filters in the Fourier domain, which are first motivated by the primitive ideas from signal processing. These filters reduce the noise by removing the high-frequency components. However, they also blur images. Stochastic modeling [70–72] originated from estimation theory has also been introduced for noise removing. In the past two decades, a number of new techniques have been introduced to improve the quality of image denoising. Statistical approaches such as maximum-likelihood estimation and adaptive (Wiener) filters are developed. Local adaptive filters are combined with impulse removal filters in the transform domain to remove not only white and mixed noise, but also their mixtures [85]. Wavelet theory [75] has been used for denoising by many researchers [78, 86–91]. The essential idea in wavelet schemes is to decompose the noisy image into a number of frequency bands, and remove the noise in appropriate frequency bands. Wavelet approaches usually perform well with a viable strategy to discriminate noise from image in each wavelet subdomain. PDE-based denoising methods were proposed to preserve image edges during the process of noise reduction as briefly reviewed in the introduction.

If impressive results are the main reasons for using nonlinear diffusion filtering in image processing, poor efficiency is the main reason for less widely spread application of nonlinear diffusion filtering in industrial codes. Finite-difference schemes are extensively used to discretize nonlinear anisotropic diffusion equations and they make the whole filtering procedure quite time consuming. We resolve this problem by using the LSEK. The efficiency of the proposed LSEK for diffusion equations has been already validated in Section 2. In the present work, we explore the performance of the LSEK image denoising. We take two different LSEK approaches.

In our first denoising approach, we choose a time-independent diffusion coefficient of the Gaussian suggested by Perona and Malik:

$$d(|\nabla u|) = \exp\left[-\frac{|\nabla u|^2}{2\sigma^2}\right], \qquad (44)$$

where the variance $\sigma$ is based on a local statistical estimate of (8). By using this variance, no additional kernel smoothing is needed. The success of this approach has been fully demonstrated in [20]. In this study, we used the proposed evolution-operator-based single-step method to obtain a fast denoising process. A Gaussian noise contaminated Lena image has been used to test the performance. It is found that the PSNR of the noisy Lena image is 21.03 db. After denoising, the quality of the Lena image has been improved and the resulting PSNR is 29.81 db. Both noisy and denoised Lena images were presented in Figure 11. We also examine our scheme for an MRI image of brain. An improvement of

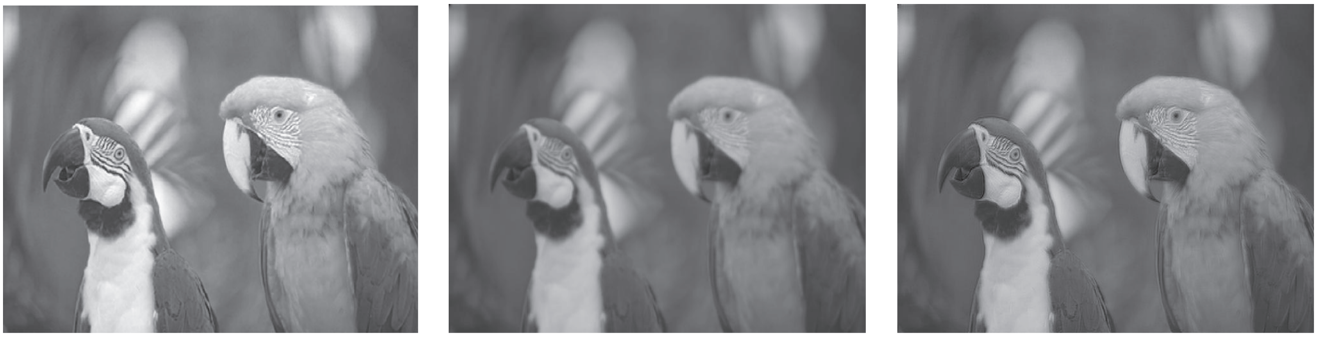

(a) (b) (c)

Figure 8: Deblurring process of the parrot image: (a) original parrot image; (b) Gaussian blurred image; (c) parrot image after deblurring.

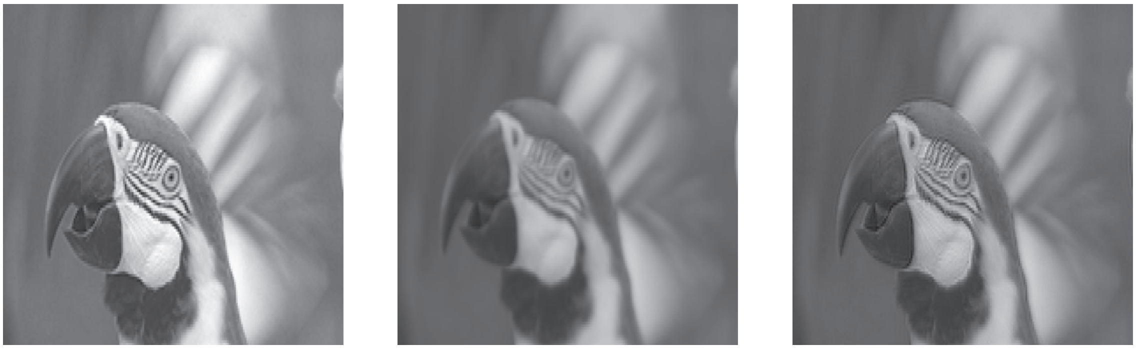

(a) (b) (c)

Figure 9: Zoomed parrot images for deblurring process: (a) original; (b) blurred; (c) processed.

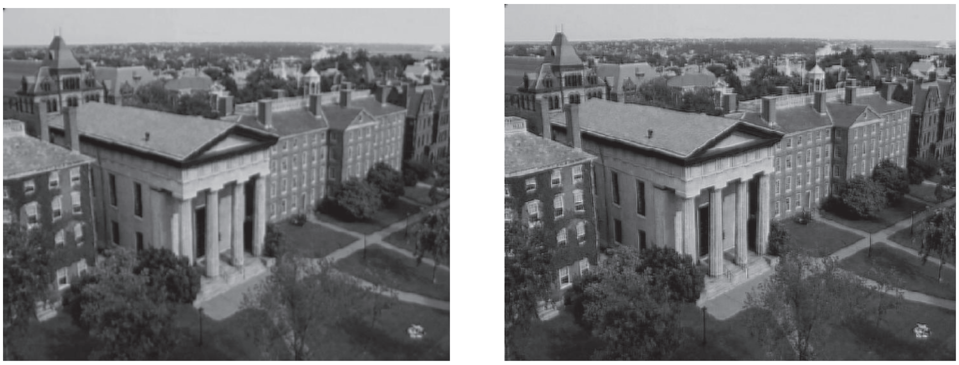

(a) (b)

Figure 10: Deblurring process of the Columbia image: (a) Gaussian blurred image; (b) Columbia image after deblurring.

6.30 db in the PSNR is obtained for this medical image, see Figure 12.

In the second approach, we use (40) directly for image denoising in a single step. From (29) and (28), it is seen that the diffusion coefficient $A_{t'}^{t} = \int_{t'}^{t} A(\tau)d\tau$ controls the local diffusion. Therefore, one can tailor $A_{t'}^{t}$ for a given purpose. In this work, we propose the following gradient-dependent $A_0^t$:

$$A_0^t(s,t) = \frac{10}{\beta}\left\{\tan^{-1}\left[s(\epsilon+\beta t)\right] - \tan^{-1}(\epsilon s)\right\}, \tag{45}$$

where $s = |G_\sigma * \nabla I|$ is the smoothed gradient magnitude, $\beta$ and $\epsilon$ are two parameters controlling the tendency of $A_0^t$. We fix $\beta = 16$ and vary $\epsilon$ between 0 and 0.1. Figure 13 shows

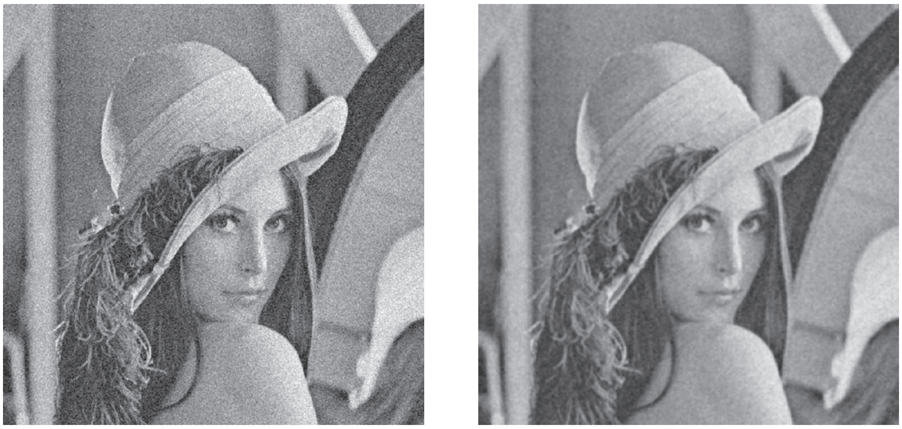

(a) (b)

Figure 11: Denoising process for the Lena image: (a) noisy Lena image with PSNR = 21.03; (b) restored Lena image with PSNR = 29.81.

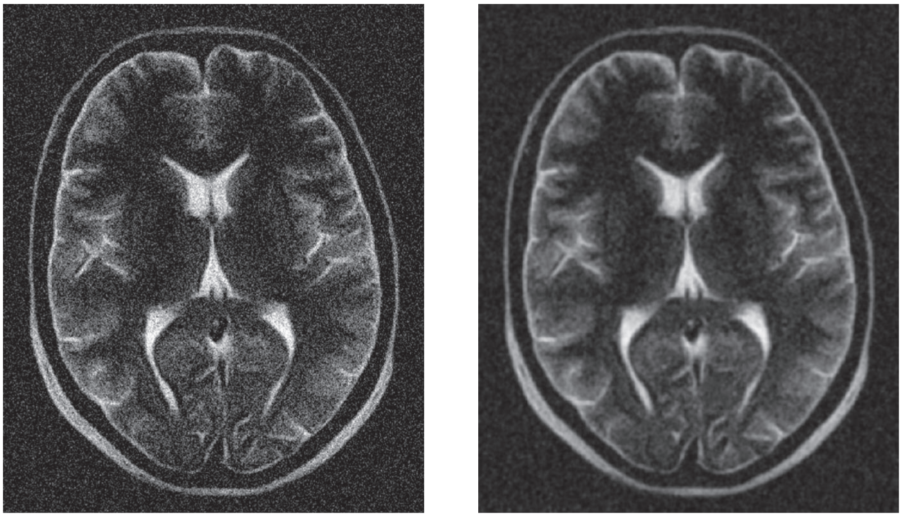

(a) (b)

Figure 12: Denoising process for the brain image: (a) noisy brain image with PSNR = 18.79; (b) restored brain image with PSNR = 25.18.

the change of $A_0^t$ with sets of $\epsilon = 0, 0.002, 0.01$, and $0.1$ at the time $t = 100$. Obviously, $\epsilon = 0$ almost corresponds to the linear diffusion process. Therefore, we set $\epsilon$ to be larger than zero in order to preserve edges of images. In our test, we take $\epsilon = 0.01$. With $\beta$ and $\epsilon$ fixed, we plot the relation between $A_0^t$ and the time $t$ in Figure 14. One can observe that $A_0^t$ basically does not change any more as the time $t$ is large enough. This implies that the denoising process is stable with respect to the stopping time. Thus, the stopping time can be chosen to be quite large without worrying about possible oversmoothing.

We classify $A_0^t$ by equally dividing the values of $A_0^t$ into $N_c = 20$ groups. We therefore generate 20 sets of LSEK coefficients accordingly. We still use the last two noisy images for our denoising demonstration, see Figure 15. The PSNR for the restored Lena image is 29.41 db and that for the restored brain image is 25.22 db. It is can be seen that the performance of this denoising procedure with a time-dependent diffusion coefficient is almost the same as that of the time-independent one. As for the stopping time, we can set it to be 5, 10, or a larger number, it is not very sensitive.

Obviously, more efficient diffusion coefficients can be designed to remove blur and noise from images, but they are beyond the scope of the present work. What we want to convey to the reader is just an efficient single-step procedure for image deblurring and denoising.

### 3.3. Image edge detection

Edges characterize object boundaries and are therefore crucial for image segmentation and registration, and for the

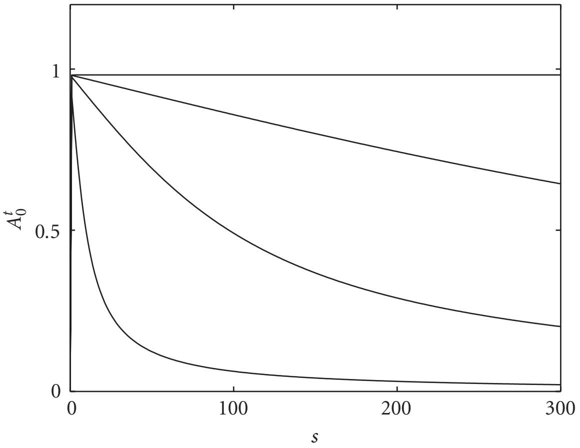

Figure 13: $A_0^t$ plotted as a function of the smoothed gradient magnitude with $\epsilon = 0, 0.002, 0.01, 0.1$ (from top to bottom); $\beta = 16$; $t = 100$.

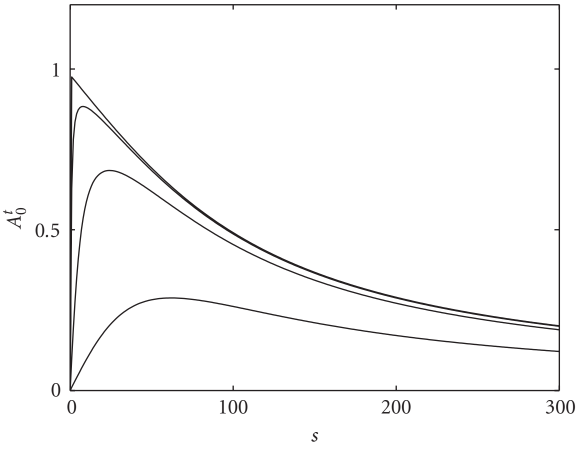

Figure 14: $A_0^t$ plotted as a function of the smoothed gradient magnitude at the times $t = 0.001$, $t = 0.01$, $t = 0.1$, and $t = 100$ (from bottom to top); $\beta = 16$; $\epsilon = 0.01$.

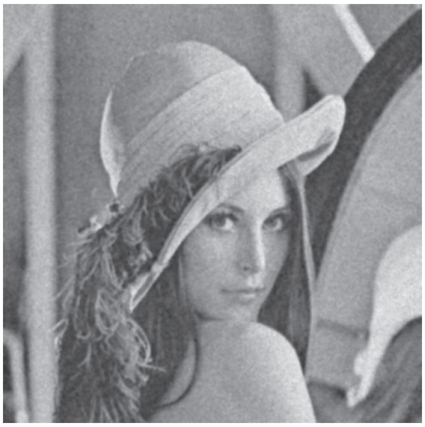

(a)

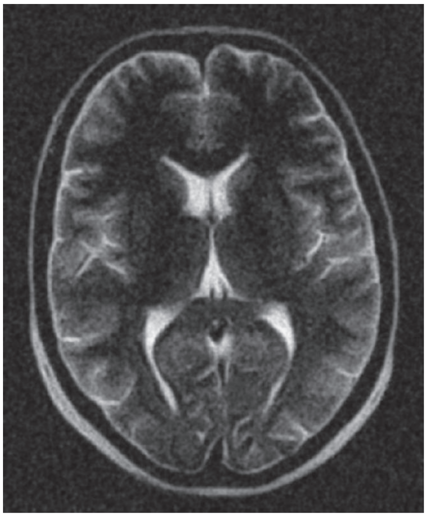

(b)

Figure 15: Time-dependent denoising process: (a) restored Lena image with PSNR = 29.41. (b) restored brain image with PSNR = 25.22.

identification of objects in scenes. In recent years, considerable research has been carried out in the development and application of efficient algorithms for the detection of the image edges. A well-known approach in the edge detection is to associate edges with zero crossing of second-order derivative. The resulting operator in use is called Laplacian of Gaussian (LoG) operator [92]. An alternative approach is the method of curve fitting [93, 94], in which an approximated function is built based on the least-square method. Therefore, a derivative on the fitting curve yields the approximate differentiation for each pixel directly. Compared with the finite-difference method, curve fitting approach results in better performance in a noisy environment. They are, however, computationally more expensive. Recently, Canny [95] has formulated edge detection as an optimization problem and defined an optimal filter which can be efficiently approximated by the first derivative of the Gaussian function. With a recursive procedure [96], the Canny detector provided an efficient way for image noise filtering and edge detection. Other edges detection methods include differentiation-based methods that use the logarithmic image processing (LIP) models [97], contrast-based methods [98], and relaxation labeling techniques [99]. In fact, these delicate techniques can achieve better performance in one way or another for common tasks. However, for images with large amount of texture, these traditional edge detection techniques are no longer reliable because of severe edge distortion. The latter is due to the wrong prediction of high-frequency edge components by low-accuracy methods. Most recently, Wei and Jia [35] proposed a novel synchronization-based realistic edge detection scheme that has demonstrated a success in detecting edges of high-texture images. In this scheme, the residual of synchronization, defined by the difference in the synchronization of two dynamic systems, gives rise to image edges as

TABLE 3: CPU-time comparison between the forward Euler scheme and the LSEK. Running the linear diffusion equation with $A = 1$ to time $t = 20$.

| Scheme | $\tau$ | CPU time(s) | Rel. $l^2$ error |
|---|---|---|---|
| Forward Euler | 0.2 | 5.01 | 0.78% |
| 2nd-order Runge-Kutta | 0.2 | 11.2 | 0.76% |
| LSEK | 20 | 0.88 | 0.15% |

if obtained by a highpass filter. However, solving two dynamic systems makes it computationally expensive. In this work, we propose a simplified version of this synchronization scheme.

The essential idea of this new edge detector is quite simple. A smoothed version (SV) of the original image $I$ is obtained by modifying the forward nonlinear diffusion operator (42). In such a nonlinear operator, the diffusion coefficient at image edges is significantly large, with contrast to the original use of nonlinear diffusion operators. As a result, image values near the edges are effectively suppressed. This smoothing operation, from the point of view of image processing, is a lowpass filter. Whereas, image edge detection should be a highpass filtering process. Therefore, edge detection version (EDV) denoted by $I_{\text{EDV}}(x, y)$ is then expressed as the difference between the original image and the smoothed image:

$$I_{\text{EDV}}(x, y) = C_1 I(x, y) - C_2 I_{\text{SV}}(x, y). \tag{46}$$

It is noted that image edges are effectively emphasized in $I_{\text{EDV}}(x, y)$ due to the edge suppressive nonlinear diffusion operator. Here $C_1$ and $C_2$ are two constants, either 1 or $-1$. We emphasize that the sign of $C_1$ and $C_2$ must be the same. If the edge of the object has a white color on a black background, we take $C_1 = C_2 = 1$. On the contrary, if the edge of the object has a black color on a white background, we choose $C_1 = C_2 = -1$. For instance, the tiger image is composed of the black object (a tiger) on the white background, $C_1$ and $C_2$ are, therefore, set to be $-1$. While the pinecone image is composed of the white object on the black background, we take $C_1 = C_2 = 1$.

Computational cost is very low in the present approach since only a single step is needed in running the nonlinear diffusion equation with our LSEK, see Table 3. Nevertheless, one can further speed up the edge detection by using a linear diffusion operator, for example, running the forward diffusion (42) till $t = 3$ by our LSEK, with a constant diffusion coefficient $A = 1$. In order to assess the computational complexity of the LSEK method, we compared in Table 4 the number of basic operations for four different methods in a single time step. These methods include the adaptive resolution scheme [41], the Perona-Malik nonlinear diffusion equation [9] resolved by the conventional explicit scheme, the AOS scheme [18] without the step of regularization, the LSEK for a linear diffusion process. Table 4 provides a quantitative analysis of computational complexity for these schemes. Multiplication or division and the addition or subtraction are calculated separately, as given in [18, 41]. In Table 4, $N$ denotes the number of pixels to treat, $m$ is the dimension of the problem considered, and $W$ is the half of the computational bandwidth. Note that the first two methods require multiple time steps, while the last two methods can be integrated in a single time step. However, the reliability of the implicit AOS scheme for a large time step needs to be validated.

We next discuss an efficient edge extraction scheme. As it is well known, thresholding is one of the most common approaches. Usually, thresholding may be viewed as an operation that tests a function $T$ of the form

$$T = T[x, y, p(x, y), I_{\text{EDV}}(x, y)], \tag{47}$$

where $p(x, y)$ denotes some local property of the point. Fortunately, experience has shown that the threshold $T$ is connected to the estimated variance which is a measure of contrast. The choice of variance can be

$$\sigma^2 = \frac{1}{2L+1}\frac{1}{2K+1}\sum_{i'=i-K}^{i+K}\sum_{j'=j-L}^{j+L}\left(I_{\text{EDV}}(i', j') - \overline{I}_{\text{EDV}_{i,j}}\right)^2, \tag{48}$$

where $\sigma^2$ denotes the local variance. Here, $\overline{I}_{\text{EDV}_{i,j}}$ represents local average and is defined as

$$\overline{I}_{\text{EDV}_{i,j}} = \frac{1}{2L+1}\frac{1}{2K+1}\sum_{i'=i-K}^{i+K}\sum_{j'=j-L}^{j+L} I_{\text{EDV}}(i', j'), \tag{49}$$

where $K$ and $L$ are the support of subimage in $x$- and $y$-directions.

The edge detector is susceptible to streaking if it uses only a single threshold. Streaking is the breaking up of an edge contour caused by fluctuation around the value of the threshold. Therefore, a more effective threshold scheme uses two thresholds $T_{\text{high}}$ and $T_{\text{low}}$ with $T_{\text{high}} \approx 2T_{\text{low}}$. In this case, streaking occurs only when it fluctuates above the high threshold and below the low threshold. Therefore, the probability of streaking is greatly reduced. The probability of isolated false edge points is also reduced because the strength of such points must be above the high threshold. We note that if the threshold $T_{\text{low}}$ is too small, there will be some false edges. Similarly, if $T_{\text{high}}$ is too large, some portion of real contour may be missing. In our scheme, the ratio of the variance of $I_{\text{EDV}}$ to that of original image is used to determine $T_{\text{low}}$, from which $T_{\text{high}}$ can be fixed, that is,

$$\begin{aligned} T_{\text{low}} &\doteq \frac{\sigma_{\text{EDI}}}{\sigma_I}\sigma_{\text{EDI}}, \\ T_{\text{high}} &\approx 2T_{\text{low}}, \end{aligned} \tag{50}$$

where $\sigma_{\text{EDI}}$ and $\sigma_I$ represent the variance of the edge detection image and that of the original image, respectively. In addition, nonmaximum suppression is performed to thin the edges in the process of edge extraction. For more detailed description, the reader is referred to [95].

To demonstrate the capabilities of the edge detector described above, we carry out experiments on various synthetic and real images, and compare the results with the output of four other detectors: Sobel detector, Prewitt detector,

Table 4: Main operations for one step of the $m$-dimensional Perona-Malik, AOS, $\lambda$-resolution, and the LSEK. (M/D: multiplications and divisions; A/S: additions and subtractions; $N$: the number of pixels; $m$: the dimensions of the problem considered; $W$: the half of the computational bandwidth.)

| Scheme | Operation | Calculate $\|\nabla I\|^2$ | $c(\|\nabla I\|)$ | Diffusion step | Total |
|---|---|---|---|---|---|
| P-M | M/D | $2mN$ | $N$ | $(2m+2)N$ | $(4m+3)N$ |
| | A/S | $(2m-1)N$ | — | $6mN$ | $(8m-1)N$ |
| AOS | M/D | $2mN$ | — | $6mN$ | $8mN$ |
| | A/S | $(2m-1)N$ | — | $6mN$ | $(8m-1)N$ |
| $\lambda$-resolution | M/D | $2mN$ | $N$ | $6\Pi$ | $(2m+7)N$ |
| | A/S | $(2m-1)N$ | — | $(2m+2)N$ | $(4m+1)N$ |
| LSEK | M/D | — | — | $(2W+1)mN$ | $(2W+1)mN$ |
| | A/S | — | — | $(2W+1)mN$ | $(2W+1)mN$ |

Canny detector, and anisotropic diffusion scheme (Perona-Malik model).

A variety of images are employed for the edge detection. We first consider the Barbara image, which is a challenging test. Figures 16(a), 16(b), 16(c), 16(d), 16(e), and 16(f) show the Barbara image and edges of the Barbara image detected by the Sobel detector, the Prewitt detector, the Canny detector, the anisotropic diffusion scheme, and the present LSEK edge detection method. It is seen that the present LSEK edge detection method is the best. Facial edges are accurately extracted and image texture is clearly detected. However, none of the other four detectors gives correct edge response to the textured part of the image. The regularity of the thin lines in the original image has been entirely distorted.

We next consider a few standard *texture images* (baboon, tiger, Goldhill, bridge, boats, and pinecone), see Figure 17. In general, the processing of texture images is a difficult task for most existing edge detection methods because the involvement of high-frequency components. For example, most existing methods are not able to distinguish baboon's bristles from the background. Moreover, it is a challenge to fully detect the thin ropes on the boats by many other existing methods. Similar fine texture, such as that in the grass and trees can be found in other four images. The ability of resolving such details is crucial to edge detection and pattern recognition. It is seen from Figure 18 that the proposed LSEK does an excellent job in the edge detection of these high-texture images. The detected image edges provide even clearer features than the original images do. For example, the pattern on the house roof and window in Goldhill image is not very clear in the original images. This feature is visible from the detected edges.

Having validated the proposed method for the edge detection of texture images, we finally apply it to a group of medical images as shown in Figure 19. The edge detection of X-ray images can be difficult because of small gradient in image resulted from small variation in X-ray attenuation, see Lung01, Lung02, and Lung04. Large amount of vessels can lead to high-texture images as shown in Lung03. Figure 20 indicates that the proposed method is able to reveal a variety of features in these images.

Based on the above discussion, it is evident that the present LSEK edge detection method is one of the best edge detectors in performance. Moreover, the present method requires only a single-time-step operation. It is these two advantages, high efficiency and excellent performance, that qualify the proposed LSEK edge detection method as one of the best approaches for image edge detection.

### 3.4. Digital image enhancement

In general, image enhancement is a process designed to increase the usefulness of the image. When images are enhanced for human viewers, the objective is to improve perceptual aspects, including image quality, intelligibility, or visual appearance. One important class of image enhancement is to modify the contrast and/or dynamic range of the image. Another class of image enhancement is to reduce the degradation of the image. In this work, we focus on the former class of issues, that is, to improve the contrast of mammograms.

A large number of investigations have been made to enhance mammography feature while reducing noise. Gordon and Rangayyan [100] used an adaptive neighborhood image processing technique to enhance the contrast of features relevant to mammography. However, this method enhanced the contrast of mammography features as well as noise. Dhawan et al. [101–103] developed an adaptive neighborhood-based image processing technique following Gordon's work. They utilized low-level analysis and knowledge in desired feature design to improve the contrast of specific features.

Filtering as an oldest technique has also been extensively used in medical imaging. For example, Tahoces et al. [104] developed a method for the enhancement of chest and breast radiographies by an automatic spatial filtering. In their work, a linear combination of an original image and two smoothed images were used. The process was completed by a nonlinear contrast stretching.

The difference image is a commonly used enhancement technique and is the basis of most existing computer-aided diagnosis systems. The difference image is usually obtained by subtracting a suppressed image from the original one to remove the background [105].

As we have discussed in the last subsection, the edge of an image is obtained based on the difference between

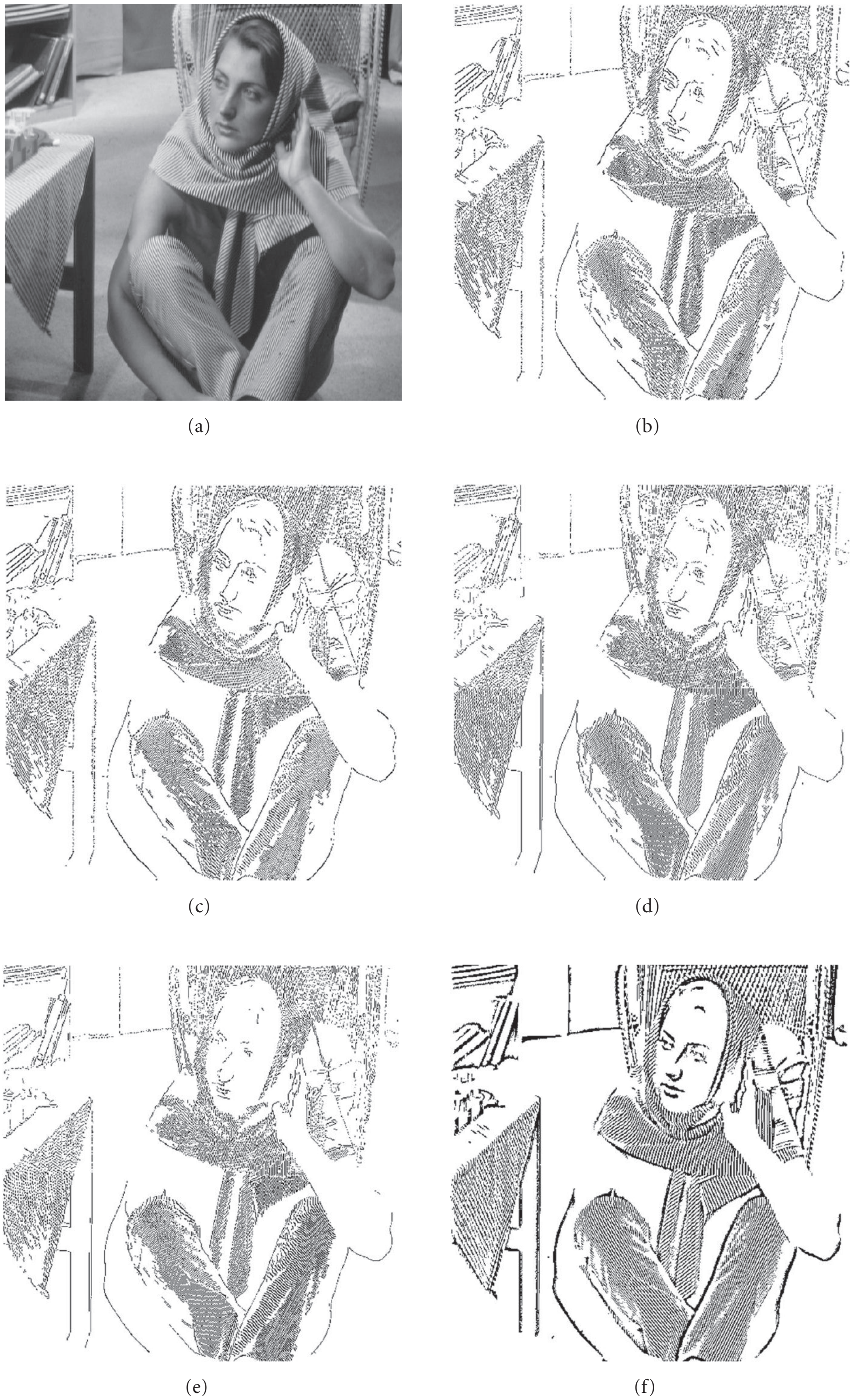

Figure 16: Edges of the Barbara image detected by different edge detectors: (a) the original Barbara image; (b) edges detected by the Sobel detector; (c) edges detected by the Prewitt detector; (d) edges detected by the Canny detector; (e) edges detected by the anisotropic diffusion scheme; (f) edges detected by the LSEK-based edge detector.

the original and the smoothed images. A similar idea is extended to image enhancement by increasing the contrast of the image. Specifically, the enhanced version (EV) of the image is produced by adding edge-detected version (EDV) to the original version:

$$\begin{aligned} I_{\mathrm{EV}}(x, y) &= I(x, y) + I_{\mathrm{EDV}}(x, y) \\ &= I(x, y) + C_3\left[I(x, y) - I_{\mathrm{SV}}(x, y)\right], \end{aligned} \tag{51}$$

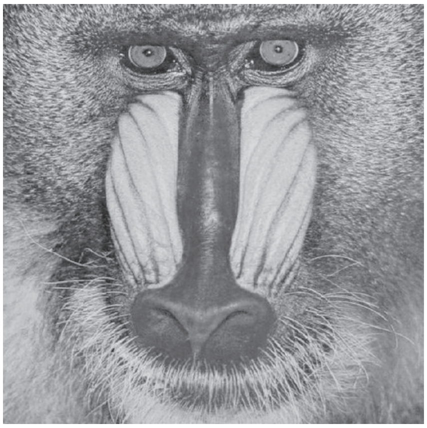

(a)

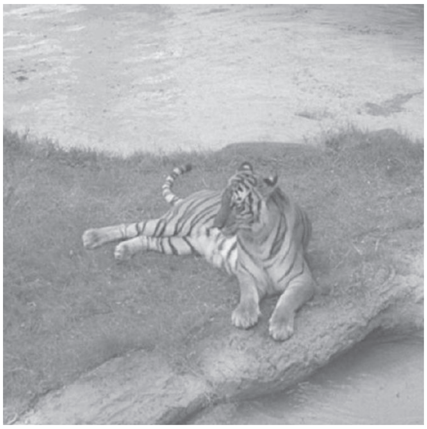

(b)

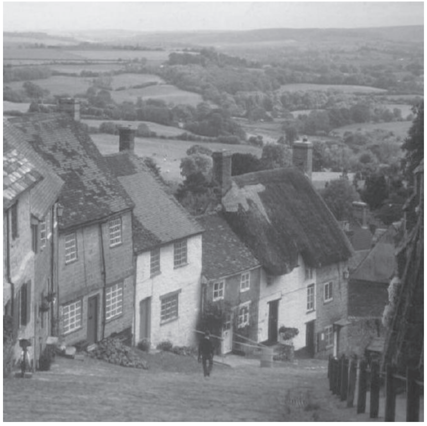

(c)

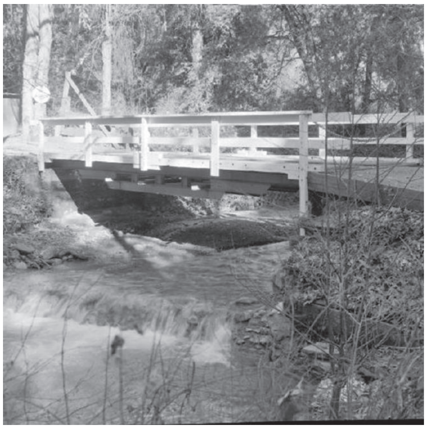

(d)

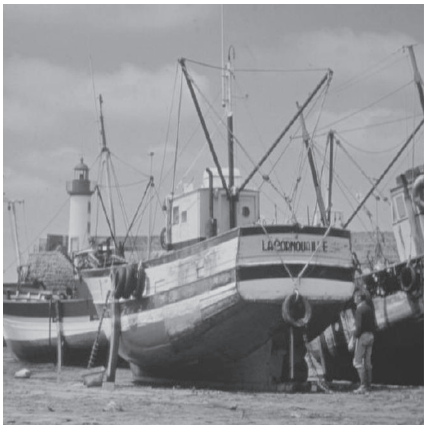

(e)

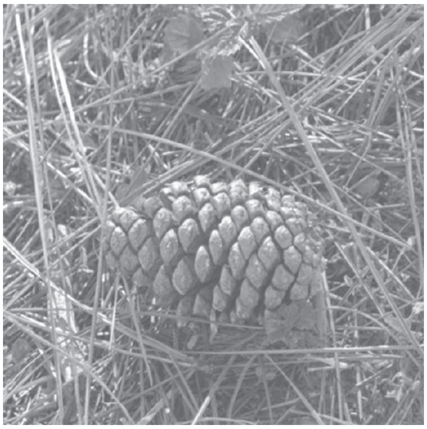

(f)

Figure 17: High-texture images: (a) baboon; (b) tiger; (c) Goldhill; (d) bridge; (e) boats; (f) pinecone.

in which the parameter $C_3$ is used to further increase the contrast between neighboring edges. There are a couple of criteria constraining the choice of the parameter $C_3$. On one hand, $C_3$ must be larger than or equal to 1. On the other hand, if $C_3$ is too large, the results may exceed the maximum and minimum values in the original version. Thus, we give another expression of the enhanced version as

$$\begin{aligned} I_{\mathrm{EV}}(x, y) &= C_4 I(x, y) + C_3 [I(x, y) - I_{\mathrm{SV}}(x, y)] \\ &= C_5 I(x, y) - C_3 I_{\mathrm{SV}}(x, y), \end{aligned} \tag{52}$$

where the parameter $C_5$ is used to control the maxima in scale-space and $C_3$ is a magnification parameter. Basically, $C_5$

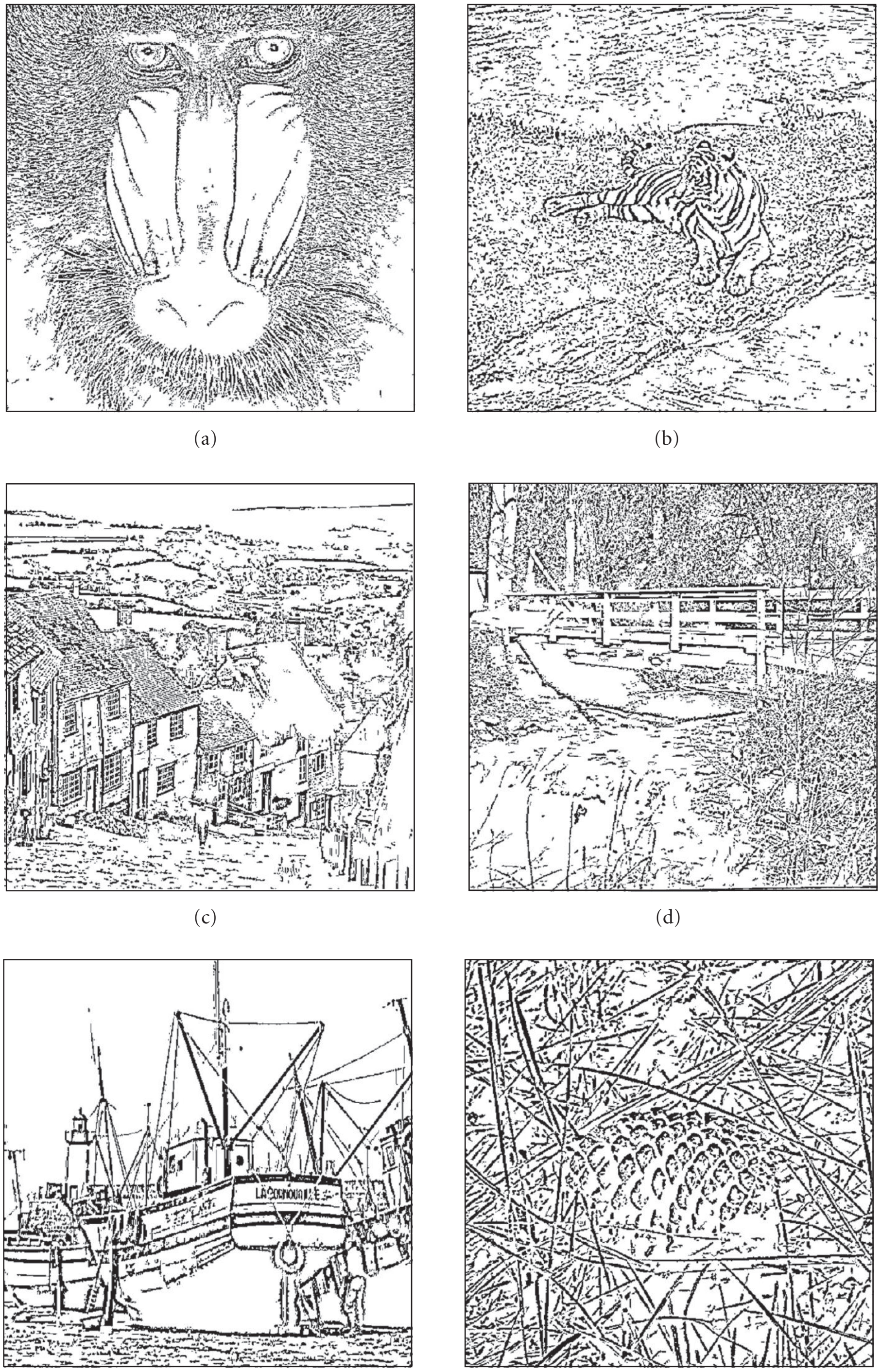

(a) (b) (c) (d) (e) (f)

Figure 18: The edges of high-texture images: (a) baboon; (b) tiger; (c) Goldhill; (d) bridge; (e) boats; (f) pinecone.

is an element of $(1, 2)$ and $C_3$ is an element of $(1, 2)$. In the above equation, the smoothed version of the image is still produced by solving the linear forward diffusion equation (42) which can be rapidly accomplished by the LSEK. The diffusion coefficient is also fixed to be $A = 1$ and the whole diffusion process is terminated at time $t = 10$.

We first consider four mammography images collected from the FTP site ftp://peipa.essex.ac.uk/ipa/pix/mias. The original images from the FTP site all have the size of $1024 \times 1024$ pixels. However, the images used in our study are cropped to the useful portion, as shown in Figure 21. The above image enhancement scheme employs two important

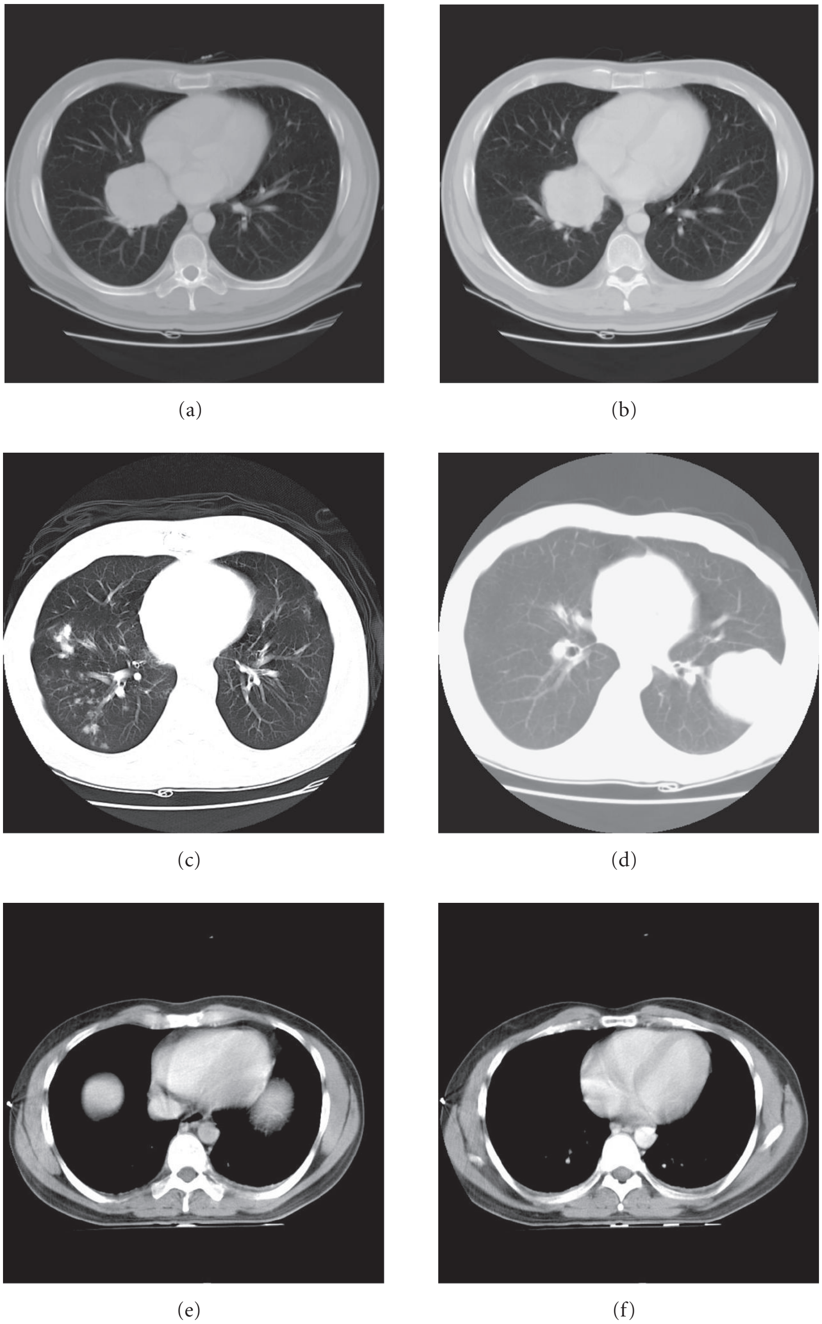

FIGURE 19: Lungs and abdomen CT images: (a) Lung01; (b) Lung02; (c) Lung03; (d) Lung04; (e) Abdomen01; (f) Abdomen02.

parameters $C_5$ and $C_3$, which are set to 1.95 and 1.05, respectively. Figure 22 shows good results for almost all test images, that is, abnormalities on all mammography images can be seen more clearly in the enhanced images than in their original ones.

We next consider four CT images of a mouse as shown in Figure 23. The same parameters used in the last example are utilized for these images. Results shown in Figure 24 indicate that the proposed method is very efficient for medical image enhancement. Nevertheless, it is worth mentioning that more attractive image enhancement results may be obtained if one chooses the diffusion equation coefficient adaptively.

### 4. CONCLUSION

Partial-differential-equation- (PDE-) based approaches have great potential for the processing of images and multidimensional data because they can be made to be systematic and automatic for large-scale high-dimensional image data.

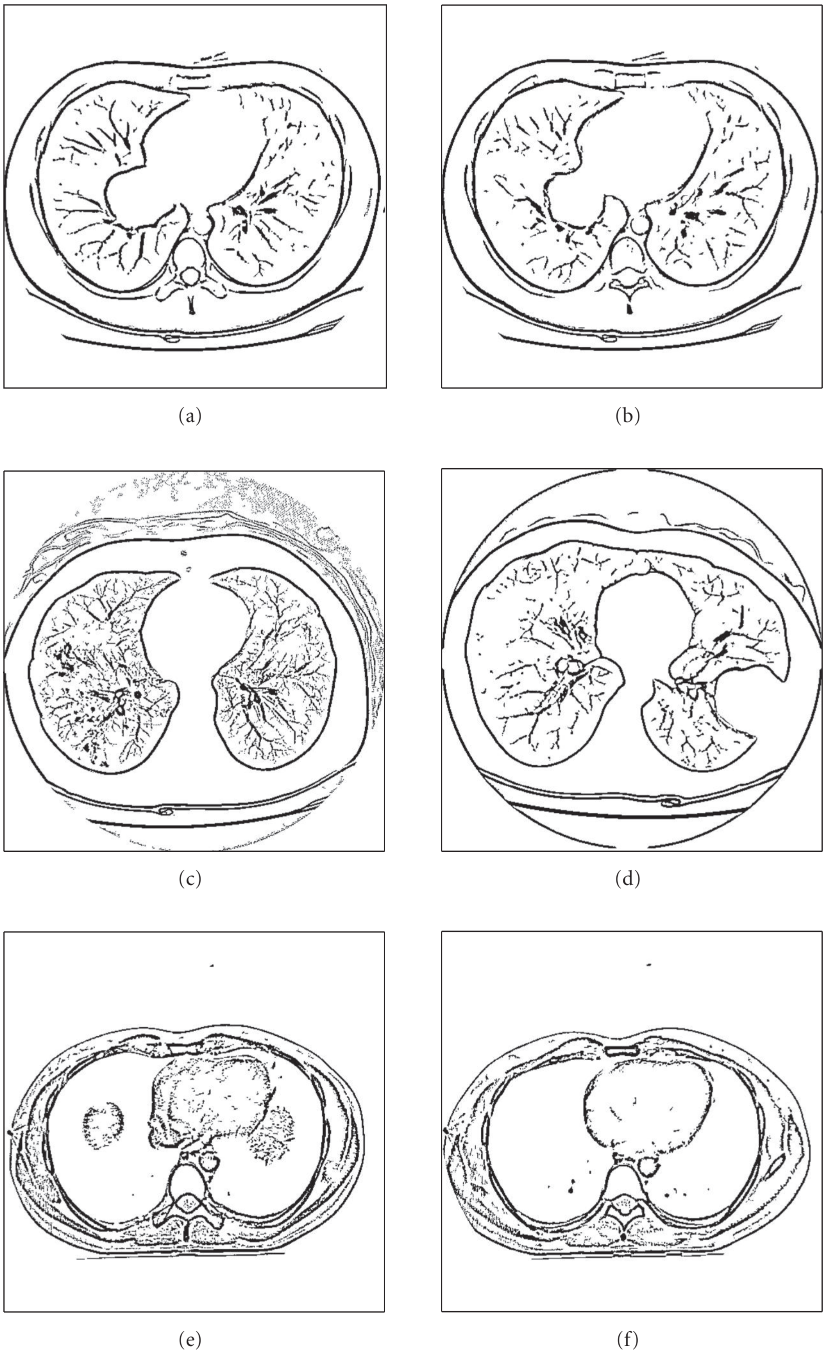

Figure 20: The edges of lungs and abdomen CT images: (a) Lung01; (b) Lung02; (c) Lung03; (d) Lung04; (e) Abdomen01; (f) Abdomen02.

Nevertheless, research in this direction is often hindered by problems in parameter optimization and extra computing time. The present work proposes an evolution-operator-based single-time-step method for image and multidimensional data processing. By utilizing a local spectral evolution kernel (LSEK) that analytically integrates a class PDEs, we show that a number of image processing operations, such as image deblurring, denoising, edge detection, and enhancement, can be effectively carried out in a single step of time integration. Filter properties of the LSEK are studied by using the Fourier analysis. Local information about the image function and its gradient is analyzed and incorporated in the time evolution of the anisotropic-type equation. In image denoising, a local-variance-based method is employed and is compared to the regularization type of approaches. Automatic exit schemes are proposed to stop image evolution in the denoising. We have achieved about 8 dB in the peak signal-to-noise improvement. In image edge detection,

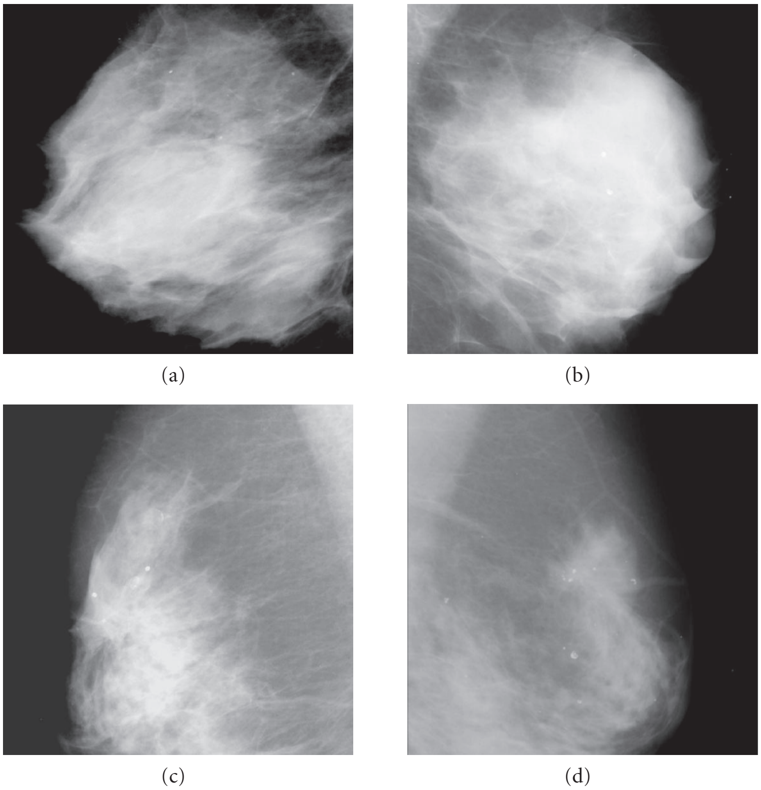

Figure 21: The mammographs used for image enhancement: (a) mdb001; (b) mdb002; (c) mdb161, (d) mdb170.

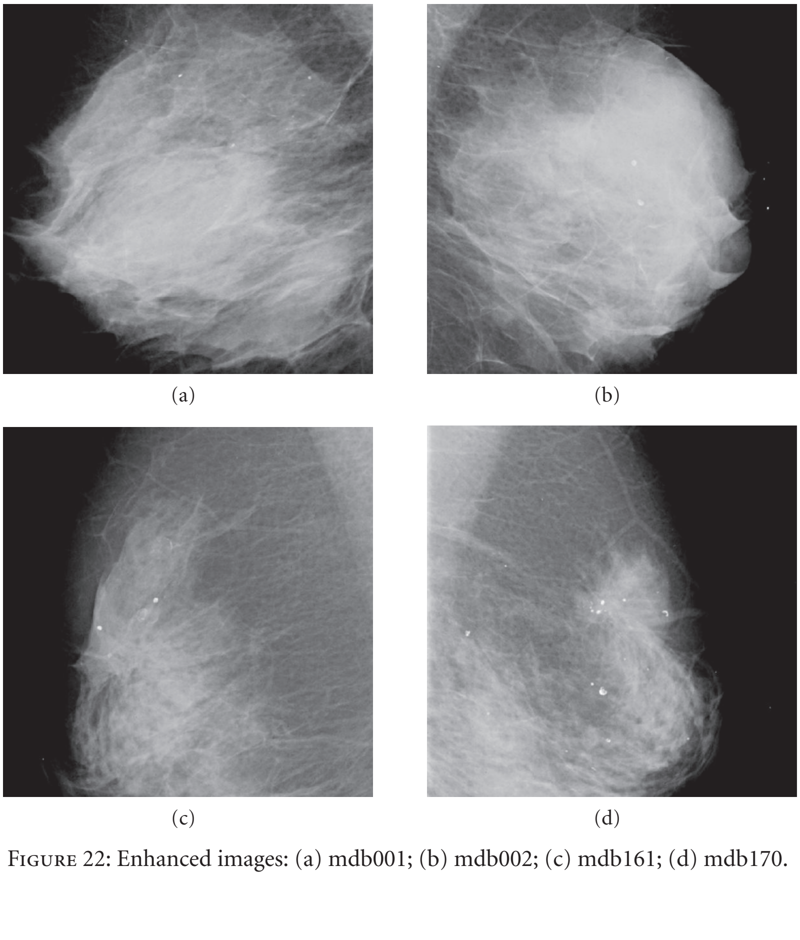

Figure 22: Enhanced images: (a) mdb001; (b) mdb002; (c) mdb161; (d) mdb170.

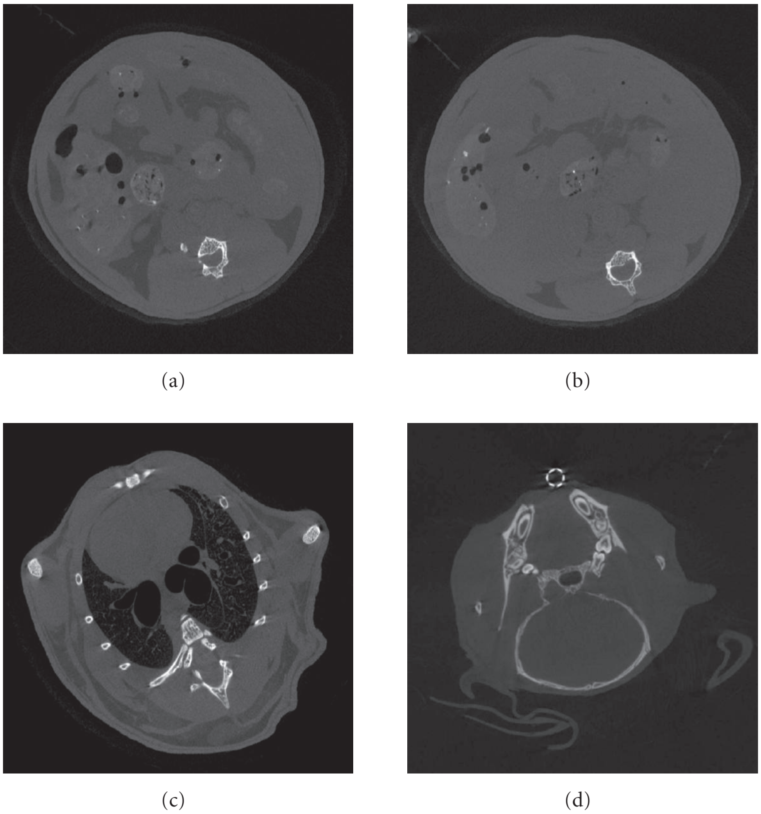

Figure 23: The CT images of mouse used for image enhancement: (a) CT001; (b) CT002; (c) CT003; (d) CT004.

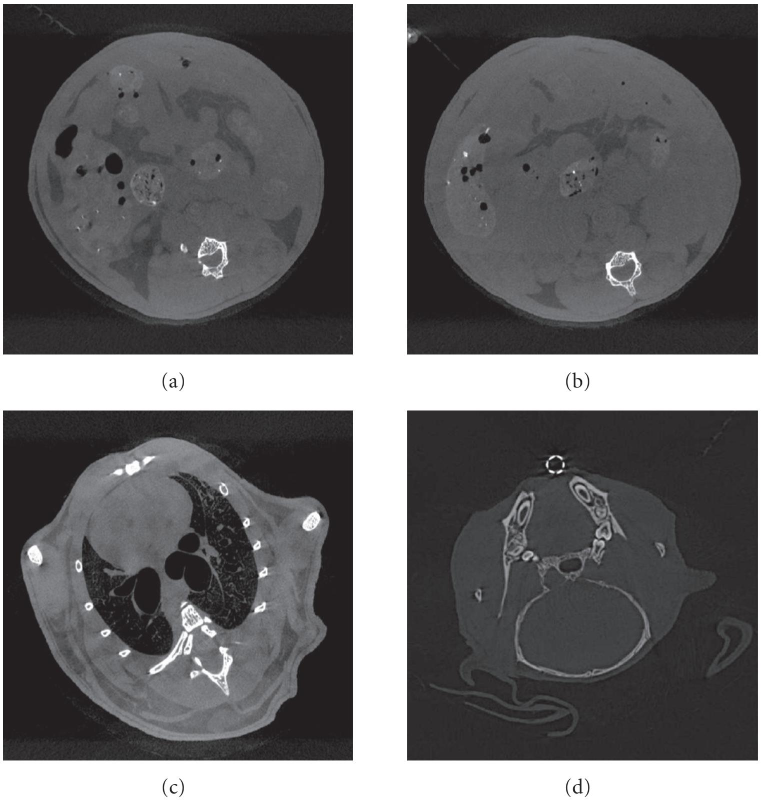

Figure 24: Enhanced images: (a) CT001; (b) CT002; (c) CT003; (d) CT004.

the proposed method utilized a simplified version of the previous coupled PDE algorithm. The present method is compared with a number of standard approaches, including Sobel, Prewitt, Canny detectors, and anisotropic diffusion operators. High-texture images and X-ray lung images are employed to demonstrate the proposed method. It is found that the proposed scheme provides some of the best performances for the edge detection of high-texture images, which is a challenge to other existing methods. Image enhancement is carried out by the combination of the image edges and the original image. Significant enhancement is achieved due to reliable image textures.

### ACKNOWLEDGMENT

This work was supported in part by NSF Grant IIS-0430987 and by IRGP Grant 71-4834.

### REFERENCES

[1] J. A. O'Sullivan, M. Jiang, X.-M. Ma, and G. Wang, "Axiomatic quantification of multidimensional image resolution," *IEEE Signal Processing Letters*, vol. 9, no. 4, pp. 120–122, 2002.

[2] A. P. Witkin, "Scale-space filtering," in *Proceedings of 8th International Joint Conference on Artificial Intelligence (IJCAI '83)*, pp. 1019–1022, Karlsruhe, West Germany, August 1983.

[3] L. Alvarez, F. Guichard, P.-L. Lions, and J.-M. Morel, "Axioms and fundamental equations of image processing," *Archive for Rational Mechanics and Analysis*, vol. 123, no. 3, pp. 199–257, 1993.

[4] M. Nielsen, P. Johansen, O. F. Olsen, and J. Weickert, Eds., *Scale-Space Theories in Computer Vision*, vol. 1682 of *Lecture Notes in Computer Science*, Springer, Berlin, Germany, 1999.

[5] R. T. Whitaker and S. M. Pizer, "A multi-scale approach to nonuniform diffusion," *CVGIP: Image Understanding*, vol. 57, no. 1, pp. 99–110, 1993.

[6] G. W. Wei and S. Zhao, "Synchronization and information processing by an on-off coupling," *Physical Review E*, vol. 65, no. 5, pp. 056210/1–056210/8, 2002.

[7] C. Goodall, "A survey of smoothing techniques," in *Modern Methods of Data Analysis*, J. Fox and J. S. Long, Eds., pp. 126–176, Sage, Newbury Park, Calif, USA, 1990.

[8] S. Zhao and G. W. Wei, "Jump process for the trend estimation of time series," *Computational Statistics & Data Analysis*, vol. 42, no. 1-2, pp. 219–241, 2003.

[9] P. Perona and J. Malik, "Scale-space and edge detection using anisotropic diffusion," *IEEE Transactions on Pattern Analysis and Machine Intelligence*, vol. 12, no. 7, pp. 629–639, 1990.

[10] M. J. Black, G. Sapiro, D. H. Marimont, and D. Heeger, "Robust anisotropic diffusion," *IEEE Transactions on Image Processing*, vol. 7, no. 3, pp. 421–432, 1998.

[11] F. Catté, P.-L. Lions, J.-M. Morel, and T. Coll, "Image selective smoothing and edge detection by nonlinear diffusion," *SIAM Journal on Numerical Analysis*, vol. 29, no. 1, pp. 182–193, 1992.

[12] S. Kichenassamy, "The Perona-Malik paradox," *SIAM Journal on Applied Mathematics*, vol. 57, no. 5, pp. 1328–1342, 1997.

[13] M. Nitzberg and T. Shiota, "Nonlinear image filtering with edge and corner enhancement," *IEEE Transactions on Pattern Analysis and Machine Intelligence*, vol. 14, no. 8, pp. 826–833, 1992.

[14] L. I. Rudin, S. Osher, and E. Fatemi, "Nonlinear total variation based noise removal algorithms," *Physica D: Nonlinear Phenomena*, vol. 60, no. 1–4, pp. 259–268, 1992.

[15] J. Shah, "A common framework for curve evolution, segmentation and anisotropic diffusion," in *Proceedings of IEEE Computer Society Conference on Computer Vision and Pattern Recognition (CVPR '96)*, pp. 136–142, San Francisco, Calif, USA, June 1996.

[16] S. Teboul, L. Blanc-Feraud, G. Aubert, and M. Barlaud, "Variational approach for edge-preserving regularization using coupled PDEs," *IEEE Transactions on Image Processing*, vol. 7, no. 3, pp. 387–397, 1998.

[17] F. Torkamani-Azar and K. E. Tait, "Image recovery using the anisotropic diffusion equation," *IEEE Transactions on Image Processing*, vol. 5, no. 11, pp. 1573–1578, 1996.

[18] J. Weickert, B. M. T. H. Romeny, and M. A. Viergever, "Efficient and reliable schemes for nonlinear diffusion filtering," *IEEE Transactions on Image Processing*, vol. 7, no. 3, pp. 398–410, 1998.

[19] Y.-L. You, W. Xu, A. Tannenbaum, and M. Kaveh, "Behavioral analysis of anisotropic diffusion in image processing," *IEEE Transactions on Image Processing*, vol. 5, no. 11, pp. 1539–1553, 1996.

[20] G. W. Wei, "Generalized Perona-Malik equation for image restoration," *IEEE Signal Processing Letters*, vol. 6, no. 7, pp. 165–167, 1999.

[21] P. V. Blomgren and T. F. Chan, "Color TV: total variation methods for restoration of vector-valued images," *IEEE Transactions on Image Processing*, vol. 7, no. 3, pp. 304–309, 1998.

[22] V. Caselles, R. Kimmel, and G. Sapiro, "Geodesic active contours," *International Journal of Computer Vision*, vol. 22, no. 1, pp. 61–79, 1997.

[23] S. Osher and L. I. Rudin, "Feature-oriented image enhancement using shock filters," *SIAM Journal on Numerical Analysis*, vol. 27, no. 4, pp. 919–940, 1990.

[24] S. Osher and L. I. Rudin, "Shocks and other nonlinear filtering applied to image processing," in *Applications of Digital Image Processing XIV*, vol. 1567 of *Proceedings of SPIE*, pp. 414–431, San Diego, Calif, USA, July 1991.

[25] G. Sapiro and D. L. Ringach, "Anisotropic diffusion of multivalued images with applications to color filtering," *IEEE Transactions on Image Processing*, vol. 5, no. 11, pp. 1582–1586, 1996.

[26] G. Sapiro, "From active contours to anisotropic diffusion: connections between basic PDE's in image processing," in *Proceedings of IEEE International Conference on Image Processing (ICIP '96)*, vol. 1, pp. 477–480, Lausanne, Switzerland, September 1996.

[27] T. F. Chan, A. Marquina, and P. Mulet, "High-order total variation-based image restoration," *SIAM Journal on Scientific Computing*, vol. 22, no. 2, pp. 503–516, 2000.

[28] Y.-L. You and M. Kaveh, "Fourth-order partial differential equations for noise removal," *IEEE Transactions on Image Processing*, vol. 9, no. 10, pp. 1723–1730, 2000.

[29] A. L. Bertozzi and J. B. Greer, "Low-curvature image simplifiers: global regularity of smooth solutions and Laplacian limiting schemes," *Communications on Pure and Applied Mathematics*, vol. 57, no. 6, pp. 764–790, 2004.

[30] J. B. Greer and A. L. Bertozzi, "Traveling wave solutions of fourth order PDEs for image processing," *SIAM Journal on Mathematical Analysis*, vol. 36, no. 1, pp. 38–68, 2004.

[31] J. B. Greer and A. L. Bertozzi, "H-1 solutions of a class of fourth order nonlinear equations for image processing,"

*Discrete and Continuous Dynamical Systems*, vol. 10, no. 1-2, pp. 349–366, 2004.
[32] M. Lysaker, A. Lundervold, and X.-C. Tai, "Noise removal using fourth-order partial differential equation with applications to medical magnetic resonance images in space and time," *IEEE Transactions on Image Processing*, vol. 12, no. 12, pp. 1579–1590, 2003.
[33] G. Gilboa, N. Sochen, and Y. Y. Zeevi, "Image sharpening by flows based on triple well potentials," *Journal of Mathematical Imaging and Vision*, vol. 20, no. 1-2, pp. 121–131, 2004.
[34] T. P. Witelski and M. Bowen, "ADI schemes for higher-order nonlinear diffusion equations," *Applied Numerical Mathematics*, vol. 45, no. 2-3, pp. 331–351, 2003.
[35] G. W. Wei and Y. Q. Jia, "Synchronization-based image edge detection," *Europhysics Letters*, vol. 59, no. 6, pp. 814–819, 2002.
[36] G. Gilboa, N. Sochen, and Y. Y. Zeevi, "Image enhancement segmentation and denoising by time dependent nonlinear diffusion processes," in *Proceedings of IEEE International Conference on Image Processing (ICIP '01)*, vol. 3, pp. 134–137, Thessaloniki, Greece, October 2001.
[37] R. Deriche, "Fast algorithms for low-level vision," *IEEE Transactions on Pattern Analysis and Machine Intelligence*, vol. 12, no. 1, pp. 78–87, 1990.
[38] T. Lindeberg, "Scale-space for discrete signals," *IEEE Transactions on Pattern Analysis and Machine Intelligence*, vol. 12, no. 3, pp. 234–254, 1990.
[39] M. Nielsen, L. Florack, and R. Deriche, "Regularization, scale-space, and edge detection filters," *Journal of Mathematical Imaging and Vision*, vol. 7, no. 4, pp. 291–307, 1997.
[40] D. Zhao and B. Li, "A new implementation of discrete multiscale filtering," in *Proceedings of IEEE International Conference on Image Processing (ICIP '96)*, vol. 1, pp. 383–385, Lausanne, Switzerland, September 1996.
[41] B. Tremblais and B. Augereau, "A fast multi-scale edge detection algorithm," *Pattern Recognition Letters*, vol. 25, no. 6, pp. 603–618, 2004.
[42] E. Bänsch and K. Mikula, "A coarsening finite element strategy in image selective smoothing," *Computing and Visualization in Science*, vol. 1, no. 1, pp. 53–61, 1997.
[43] J. Kačur and K. Mikula, "Solution of nonlinear diffusion appearing in image smoothing and edge detection," *Applied Numerical Mathematics*, vol. 17, no. 1, pp. 47–59, 1995.
[44] T. Preußer and M. Rumpf, "An adaptive finite element method for large scale image processing," in *Scale-Space Theories in Computer Vision*, M. Nielsen, P. Johansen, O. F. Olsen, and J. Weickert, Eds., vol. 1682 of *Lecture Notes in Computer Science*, pp. 223–234, Springer, Berlin, Germany, 1999.
[45] Z. Krivá and K. Mikula, "An adaptive finite volume scheme for solving nonlinear diffusion equations in image processing," *Journal of Visual Communication and Image Representation*, vol. 13, no. 1-2, pp. 22–35, 2002.
[46] K. Mikula and N. Ramarosy, "Semi-implicit finite volume scheme for solving nonlinear diffusion equations in image processing," *Numerische Mathematik*, vol. 89, no. 3, pp. 561–590, 2001.
[47] J. Fröhlich and J. Weickert, "Image processing using a wavelet algorithm for nonlinear diffusion," Tech. Rep. 104, Laboratory of Technomathematics, University of Kaiserslautern, Kaiserslautern, Germany, March 1994.
[48] F. L. Fontaine and S. Basu, "Wavelet-based solution to anisotropic diffusion equation for edge detection," *International Journal of Imaging Systems and Technology*, vol. 9, no. 5, pp. 356–368, 1998.
[49] S. T. Acton, A. C. Bovik, and M. M. Crawford, "Anisotropic diffusion pyramids for image segmentation," in *Proceedings of IEEE International Conference on Image Processing (ICIP '94)*, vol. 3, pp. 478–482, Austin, Tex, USA, November 1994.
[50] J. A. Sethian, *Level Set Methods: Evolving Interfaces in Geometry, Fluid Mechanics, Computer Vision, and Materials Science*, Cambridge University Press, Cambridge, UK, 1996.
[51] K. Siddiqi, B. B. Kimia, and C.-W. Shu, "Geometric shock-capturing ENO schemes for subpixel interpolation, computation and curve evolution," *Graphical Models and Image Processing*, vol. 59, no. 5, pp. 278–301, 1997.
[52] B. Jawerth, P. Lin, and E. Sinzinger, "Lattice Boltzmann models for anisotropic diffusion of images," *Journal of Mathematical Imaging and Vision*, vol. 11, no. 3, pp. 231–237, 1999.
[53] U. S. Ranjan and K. R. Ramakrishnan, "A stochastic scale space for multiscale image representation," in *Scale-Space Theories in Computer Vision*, M. Nielsen, P. Johansen, O. F. Olsen, and J. Weickert, Eds., vol. 1682 of *Lecture Notes in Computer Science*, pp. 441–446, Springer, Berlin, Germany, 1999.
[54] G. W. Wei, "Discrete singular convolution for the solution of the Fokker-Planck equation," *The Journal of Chemical Physics*, vol. 110, no. 18, pp. 8930–8942, 1999.
[55] G. Bao, G. W. Wei, and S. Zhao, "Local spectral time-domain method for electromagnetic wave propagation," *Optics Letters*, vol. 28, no. 7, pp. 513–515, 2003.
[56] Z. J. Hou and G. W. Wei, "A new approach to edge detection," *Pattern Recognition*, vol. 35, no. 7, pp. 1559–1570, 2002.
[57] Z. Shao, G. W. Wei, and S. Zhao, "DSC time-domain solution of Maxwell's equations," *Journal of Computational Physics*, vol. 189, no. 2, pp. 427–453, 2003.
[58] G. W. Wei, "Discrete singular convolution for the sine-Gordon equation," *Physica D: Nonlinear Phenomena*, vol. 137, no. 3-4, pp. 247–259, 2000.
[59] G. W. Wei, "Vibration analysis by discrete singular convolution," *Journal of Sound and Vibration*, vol. 244, no. 3, pp. 535–553, 2001.
[60] G. W. Wei, Y. B. Zhao, and Y. Xiang, "The determination of natural frequencies of rectangular plates with mixed boundary conditions by discrete singular convolution," *International Journal of Mechanical Sciences*, vol. 43, no. 8, pp. 1731–1746, 2001.
[61] S. Zhao and G. W. Wei, "Comparison of the discrete singular convolution and three other numerical schemes for solving Fisher's equation," *SIAM Journal on Scientific Computing*, vol. 25, no. 1, pp. 127–147, 2003.
[62] Y. C. Zhou and G. W. Wei, "High resolution conjugate filters for the simulation of flows," *Journal of Computational Physics*, vol. 189, no. 1, pp. 159–179, 2003.
[63] Y. C. Zhou, B. S. V. Patnaik, D. C. Wan, and G. W. Wei, "DSC solution for flow in a staggered double lid driven cavity," *International Journal for Numerical Methods in Engineering*, vol. 57, no. 2, pp. 211–234, 2003.
[64] S. N. Yu, S. Zhao, and G. W. Wei, "Local spectral time splitting method for first- and second-order partial differential equations," *Journal of Computational Physics*, vol. 206, no. 2, pp. 727–780, 2005.
[65] P. Lazaridis, G. Debarge, and P. Gallion, "Split-step-Gauss-Hermite algorithm for fast and accurate simulation of soliton propagation," *International Journal of Numerical Modelling: Electronic Networks, Devices and Fields*, vol. 14, no. 4, pp. 325–329, 2001.

[66] J. Korevaar, "Pansions and the theory of Fourier transforms," *Transactions of the American Mathematical Society*, vol. 91, no. 1, pp. 53–101, 1959.
[67] D. K. Hoffman, N. Nayar, O. A. Sharafeddin, and D. J. Kouri, "Analytic banded approximation for the discretized free propagator," *Journal of Physical Chemistry*, vol. 95, no. 21, pp. 8299–8305, 1991.
[68] M. Jiang and G. Wang, "Convergence of the simultaneous algebraic reconstruction technique (SART)," *IEEE Transactions on Image Processing*, vol. 12, no. 8, pp. 957–961, 2003.
[69] M. Jiang and G. Wang, "Convergence studies on iterative algorithms for image reconstruction," *IEEE Transactions on Medical Imaging*, vol. 22, no. 5, pp. 569–579, 2003.
[70] C. Bouman and K. Sauer, "A generalized Gaussian image model for edge-preserving MAP estimation," *IEEE Transactions on Image Processing*, vol. 2, no. 3, pp. 296–310, 1993.
[71] G. Demoment, "Image reconstruction and restoration: overview of common estimation structures and problems," *IEEE Transactions on Acoustics, Speech, and Signal Processing*, vol. 37, no. 12, pp. 2024–2036, 1989.
[72] S. Geman and D. Geman, "Stochastic relaxation, Gibbs distributions, and the Bayesian restoration of images," *IEEE Transactions on Pattern Analysis and Machine Intelligence*, vol. 6, no. 6, pp. 721–741, 1984.
[73] A. K. Jain, *Fundamentals of Digital Image Processing*, Prentice-Hall, Englewood Cliffs, NJ, USA, 1989.
[74] M. Jiang, G. Wang, M. W. Skinner, J. T. Rubinstein, and M. W. Vannier, "Blind deblurring of spiral CT images," *IEEE Transactions on Medical Imaging*, vol. 22, no. 7, pp. 837–845, 2003.
[75] S. G. Mallat, "Multifrequency channel decompositions of images and wavelet models," *IEEE Transactions on Acoustics, Speech, and Signal Processing*, vol. 37, no. 12, pp. 2091–2110, 1989.
[76] M. R. Banham, N. P. Galatsanos, H. L. Gonzalez, and A. K. Katsaggelos, "Multichannel restoration of single channel images using a wavelet-based subband decomposition," *IEEE Transactions on Image Processing*, vol. 3, no. 6, pp. 821–833, 1994.
[77] P. Charbonnier, L. Blanc-Féraud, and M. Barlaud, "Noisy image restoration using multiresolution Markov random fields," *Journal of Visual Communication and Image Representation*, vol. 3, no. 4, pp. 338–346, 1992.
[78] D. L. Donoho and I. M. Johnstone, "Adapting to unknown smoothness via wavelet shrinkage," *Journal of the American Statistical Association*, vol. 90, no. 432, pp. 1200–1224, 1995.
[79] J.-L. Starck and A. Bijaoui, "Filtering and deconvolution by the wavelet transform," *Signal Processing*, vol. 35, no. 3, pp. 195–211, 1994.
[80] Y. Y. Li and F. Santosa, "A computational algorithm for minimizing total variation in image restoration," *IEEE Transactions on Image Processing*, vol. 5, no. 6, pp. 987–995, 1996.
[81] D. C. Dobson and C. R. Vogel, "Convergence of an iterative method for total variation denoising," *SIAM Journal on Numerical Analysis*, vol. 34, no. 5, pp. 1779–1791, 1997.
[82] T. F. Chan, G. H. Golub, and P. Mulet, "A nonlinear primal-dual method for total variation-based image restoration," *SIAM Journal on Scientific Computing*, vol. 20, no. 6, pp. 1964–1977, 1999.
[83] J. F. Meinel Jr., G. Wang, M. Jiang, T. Frei, M. W. Vannier, and E. A. Hoffman, "Spatial variation of resolution and noise in multi-detector row spiral CT," *Academic Radiology*, vol. 10, no. 6, pp. 607–613, 2003.
[84] J. Astola and P. Kuosmanen, *Fundamentals of Nonlinear Digital Filtering*, CRC Press LLC, Boca Raton, Fla, USA, 1997.
[85] K. Egiazarian, J. Astola, M. Helsingius, and P. Kuosmanen, "Adaptive denoising and lossy compression of images in transform domain," *Journal of Electronic Imaging*, vol. 8, no. 3, pp. 233–245, 1999.
[86] S. G. Chang, B. Yu, and M. Vetterli, "Adaptive wavelet thresholding for image denoising and compression," *IEEE Transactions on Image Processing*, vol. 9, no. 9, pp. 1532–1546, 2000.
[87] R. R. Coifman and D. L. Donoho, "Translation-invariant denoising," in *Wavelets and Statistics*, vol. 103 of *Lecture Notes in Statistics*, pp. 125–150, Springer, Heidelberg, Germany, 1995.
[88] R. T. Ogden, *Essential Wavelets for Statistical Applications and Data Analysis*, Birkhäuser Boston, Boston, Mass, USA, 1997.
[89] Z. Shi, G. W. Wei, D. J. Kouri, D. K. Hoffman, and Z. Bao, "Lagrange wavelets for signal processing," *IEEE Transactions on Image Processing*, vol. 10, no. 10, pp. 1488–1508, 2001.
[90] B. Vidakovic, *Statistical Modeling by Wavelets*, Wiley Series in Probability and Statistics, John wiley & Sons, New York, NY, USA, 1999.
[91] J. B. Weaver, Y. S. Xu, D. M. Healy Jr., and L. D. Cromwell, "Filtering noise from images with wavelet transforms," *Magnetic Resonance in Medicine*, vol. 21, no. 2, pp. 288–295, 1991.
[92] D. Marr and E. Hildreth, "Theory of edge detection," *Proceedings of the Royal Society of London. Series B, Biological Sciences*, vol. 207, no. 1167, pp. 187–217, 1980.
[93] R. M. Haralick and L. T. Watson, "A facet model for image data," *Computer Graphics and Image Processing*, vol. 15, no. 2, pp. 113–129, 1981.
[94] V. S. Nalwa and T. O. Binford, "On detecting edges," *IEEE Transactions on Pattern Analysis and Machine Intelligence*, vol. 8, no. 6, pp. 699–714, 1986.
[95] J. Canny, "A computational approach to edge detection," *IEEE Transactions on Pattern Analysis and Machine Intelligence*, vol. 8, no. 6, pp. 679–698, 1986.
[96] R. Deriche, "Optimal edge detection using recursive filtering," in *Proceedings of 1st IEEE International Conference on Computer Vision (ICCV '87)*, pp. 501–505, London, UK, June 1987.
[97] G. Deng and J.-C. Pinoli, "Differentiation-based edge detection using the logarithmic image processing model," *Journal of Mathematical Imaging and Vision*, vol. 8, no. 2, pp. 161–180, 1998.
[98] R. P. Johnson, "Contrast based edge detection," *Pattern Recognition*, vol. 23, no. 3-4, pp. 311–318, 1990.
[99] S. S. Iyengar and W. Deng, "An efficient edge detection algorithm using relaxation labeling technique," *Pattern Recognition*, vol. 28, no. 4, pp. 519–536, 1995.
[100] R. Gordon and R. M. Rangayyan, "Feature enhancement of film mammograms using fixed and adaptive neighborhoods," *Applied Optics*, vol. 23, no. 4, pp. 560–564, 1984.
[101] A. P. Dhawan, G. Buelloni, and R. Gordon, "Enhancement of mammographic features by optimal adaptive neighborhood image processing," *IEEE Transactions on Medical Imaging*, vol. MI-5, no. 1, pp. 8–15, 1986.
[102] A. P. Dhawan and R. Gordon, "Reply to comments on enhancement of mammographic features by optimal adaptive neighborhood image processing," *IEEE Transactions on Medical Imaging*, vol. MI-6, no. 1, pp. 82–83, 1987.
[103] A. P. Dhawan and E. Le Royer, "Mammographic feature enhancement by computerized image processing," *Computer Methods and Programs in Biomedicine*, vol. 27, no. 1, pp. 23–35, 1988.
[104] P. G. Tahoces, J. Correa, M. Souto, C. Gonzalez, L. Gomez, and J. J. Vidal, "Enhancement of chest and breast radiographs by automatic spatial filtering," *IEEE Transactions on Medical Imaging*, vol. 10, no. 3, pp. 330–335, 1991.

[105] H. P. Chan, K. Doi, C. J. Vyborny, K. L. Lam, and R. A. Schmidt, "Computer-aided detection of microcalcifications in mammograms. Methodology and preliminary clinical study," *Investigative Radiology*, vol. 23, no. 9, pp. 664–671, 1988.

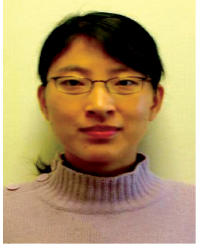

**Yuhui Sun** received her B.S. degree in mechanical engineering from Northwestern Polytechnical University, Xi'an, China, in 1998. She is currently pursuing the Ph.D. degree in applied mathematics at the Department of Mathematics, Michigan State University. Her main research interests are related to PDE-based methods for image denoising, enhancement, edge detection, and segmentation. Her other research interests include scientific computing and computational fluid dynamics.

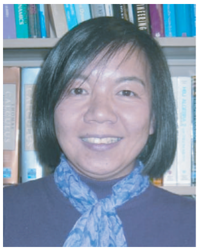

**Peiru Wu** received Ph.D. degree in computational fluid dynamics from Massachusetts Institute of Technology in 1991. Her Postdoctoral research emphasized mathematical model and simulation of the proteins behavior in Department of Chemical Engineering at University of Delaware. Subsequently, she worked as a Researcher Associate at Department of Chemistry at Michigan State University(MSU). Later, she worked on data mining, scientific computing, and bioinformatics in Pfizer until returning back to MSU as a faculty member in Department of Mathematics in 2002. Her current research interests include bioinformatics, biomedical image processing, mathematical biology, and industrial mathematics. She is the coordinator of the Professional Master of Science (proMSc) Industrial Mathematics Program at Michigan State University. She has extensive experience in both academia and industry, and she has a great deal of experience on data mining analysis by using statistics and other advanced methods, and developing data mining algorithms.

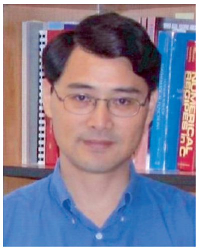

**G. W. Wei** received his Ph.D. degree from the University of British Columbia in 1996. With a Postdoctoral Fellowship Award from the Natural Sciences and Engineering Research Council (NSERC) of Canada, he did research work at the University of Houston, where he was appointed as a Research Assistant Professor in 1997. In 1998, he joined the faculty of the National University of Singapore and was promoted to Associate Professor in 2001. In 2002, he relocated to Michigan State University, where he is currently an Associate Professor of mathematics, and electrical and computer engineering. He has advised 8 doctoral students, 7 master students, and 8 postdoctoral fellows. He has authored or coauthored over 100 refereed journal papers. His current research interests include scientific and engineering computations, biomedical image analysis, controlling chaos and turbulence, mathematical modeling, and computation of protein structures and interactions.

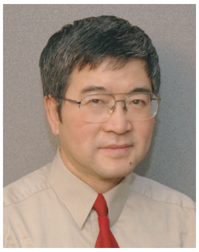

**Ge Wang** received B.E. degree in electrical engineering from Xidian University, X'ian, China, in 1982, M.S. degree in remote sensing from Graduate School of Academia Sinica, Beijing, China, in 1985, and M.S. and Ph.D. degrees in electrical and computer engineering from State University of New York, Buffalo, in 1991 and 1992. He was Instructor and Assistant Professor with Department of Electrical Engineering, Graduate School of Academia Sinica, from 1984 to 1988, Instructor and Assistant Professor with Mallinckrodt Institute of Radiology, Washington University, St. Louis, Mo, from 1992 to 1996. He was Associate Professor with University of Iowa from 1997 to 2002. Currently, he is Professor with Departments of Radiology, Biomedical Engineering, Civil Engineering, and Mathematics, and Director of the Center for X-Ray and Optical Tomography, University of Iowa. His interests include computed tomography, bioluminescence tomography, and systems biomedicine. He has authored or coauthored over 300 journal articles and conference papers, including the first paper on spiral/helical cone-beam CT and the first paper on bioluminescence tomography. He is the Editor-in-Chief for International Journal of Biomedical Imaging, and Associate Editor for IEEE Transactions Medical, and Associate Editor for IEEE Transactions on Medical Imaging and Medical Physics. He is an IEEE Fellow and an AIMBE Fellow. He is also recognized by a number of awards for academic achievements.

